# *Propionibacterium* spp.—source of propionic acid, vitamin B12, and other metabolites important for the industry

**DOI:** 10.1007/s00253-017-8616-7

**Published:** 2017-11-22

**Authors:** Kamil Piwowarek, Edyta Lipińska, Elżbieta Hać-Szymańczuk, Marek Kieliszek, Iwona Ścibisz

**Affiliations:** 1 Department of Biotechnology, Microbiology and Food Evaluation, Division of Food Biotechnology and Microbiology, Faculty of Food Sciences, Warsaw University of Life Sciences SGGW (WULS-SGGW), Nowoursynowska 159c Street, 02-776 Warsaw, Poland; 2 Department of Food Technology, Division of Fruit and Vegetable Technology, Faculty of Food Sciences, Warsaw University of Life Sciences (WULS-SGGW), Nowoursynowska 159c Street, 02-776 Warsaw, Poland

**Keywords:** *Propionibacterium*, Propionic acid, Vitamin B12, Trehalose, Bacteriocins

## Abstract

Bacteria from the *Propionibacterium* genus consists of two principal groups: cutaneous and classical. Cutaneous *Propionibacterium* are considered primary pathogens to humans, whereas classical *Propionibacterium* are widely used in the food and pharmaceutical industries. Bacteria from the *Propionibacterium* genus are capable of synthesizing numerous valuable compounds with a wide industrial usage. Biomass of the bacteria from the *Propionibacterium* genus constitutes sources of vitamins from the B group, including B12, trehalose, and numerous bacteriocins. These bacteria are also capable of synthesizing organic acids such as propionic acid and acetic acid. Because of GRAS status and their health-promoting characteristics, bacteria from the *Propionibacterium* genus and their metabolites (propionic acid, vitamin B12, and trehalose) are commonly used in the cosmetic, pharmaceutical, food, and other industries. They are also used as additives in fodders for livestock. In this review, we present the major species of *Propionibacterium* and their properties and provide an overview of their functions and applications. This review also presents current literature concerned with the possibilities of using *Propionibacterium* spp. to obtain valuable metabolites. It also presents the biosynthetic pathways as well as the impact of the genetic and environmental factors on the efficiency of their production.

## Introduction

Till date, numerous studies have been conducted regarding the use of the bacteria from *Propionibacterium* genus, which revealed, among others, that these bacteria are capable of biosynthesizing valuable metabolites, such as propionic acid, vitamin B12, bacteriocins, and trehalose. This suggests that they constitute an important group of microorganisms that are industrially important in the future. The major advantage of bacteria from the *Propionibacterium* genus is that they have the capacity to grow and synthesize metabolites on substrates containing different industrial waste products, which considerably elevates the economic profitability of biotechnological processes (Huang et al. 2002; Yazdani and Gonzales 2007; Zhu et al. 2010; Feng et al. 2011; Ruhal and Choudhury 2012a **;** Zhu et al. 2012; Wang and Yang 2013; Piwowarek et al. 2016 **)**. Bacteria from the *Propionibacterium* genus and their metabolites (propionic acid, vitamin B12, and trehalose) are commonly used in the cosmetic, pharmaceutical, and food industries. They are also used as additives in fodders for livestock. In this study, we present the most recent literature review regarding the bacteria of the *Propionibacterium* genus and their metabolites such as propionic acid, vitamin B12, trehalose, and all of the bacteriocins known and their current and potential use in different industries (Thierry et al. 2005; Lee et al. 2013; Cousin et al. 2016; Divek and Kollanoor-Johny 2016; Angelopoulou et al. 2017). Moreover, the biosynthetic pathways of these metabolites and the influence of environmental and genetic factors (Falentin et al. 2010) on the efficiency of these processes and the impact of different industrial waste products as carbon sources on the biosynthesis of these metabolites are reviewed.

## Characterization of *Propionibacterium*

Bacteria from the *Propionibacterium* genus were isolated and described in the first half of the twentieth century by Eduard von Freudenreich, Orl-Jensen, and van Niela, who classified this genus into class *Actinobacteria*, order *Actinomycetales*, and family *Propionibacteriaceae* (Breed et al. 1957). Bacteria from the *Propionibacterium* genus are divided into two groups based on their habitat: skin (acnes) and classical (dairy). The first group comprises species that are present on the human skin and in the oral and the gastrointestinal mucosa, such as *Propionibacterium acnes*, *Propionibacterium avidum*, *Propionibacterium propionicum*, *Propionibacterium granulosum*, and *Propionibacterium lymphophilum* (all these are pathogenic microorganisms). Microorganisms belonging to the second phylogenetic group include the classical strains: the first group comprises bacteria from *Propionibacterium acidipropionici*, *Propionibacterium jensenii*, and *Propionibacterium thoenii* species; the second group contains subspecies within *Propionibacterium freudenreichii* (subsp. *shermanii*, subsp. *freudenreichii*) (Meile et al. 1999). These subspecies vary with respect to two features: ability to reduce nitrates and ability to metabolize lactose. Bacterial strains from *P. freudenreichii* subsp. *freudenreichii* can reduce nitrates, but they do not have the ability of lactose fermentation. However, strains of *P. freudenreichii* subsp*. shermanii* can metabolize lactose (they have genes encoding β-D galactosidase enzyme - EC 3.2.1.23), but they are not capable of reducing nitrates. All classical bacteria from the *Propionibacterium* genus have fermentation capability, and they are major sources of valuable metabolites, such as propionic acid, vitamin B12, bacteriocin, and trehalose. Propionic acid bacteria (PAB) are used in the production of cheese (vaccine components for Swiss cheeses and Swiss-style Dutch cheeses), pickle, silage, and as probiotics in animal nutrition. Metabolites obtained from PAB are used as preservatives. *Propionibacterium* spp. are present on the herbaceous plants and in the rumen of the bovine species, excrements of the herbivores, soil, sewage, sludge, milk, pickle, water after oil production, and in fermented orange juice (Kusano et al. 1997; Meile et al. 1999; Koussémon et al. 2003; Leverrier et al. 2004; Suomalainen et al. 2008).

*Propionibacterium* spp. are Gram-positive bacilli, which means, they are nonmotile and do not produce bacterial spores, are catalase-positive, and have a length of 1–5 μm. They are recognized as either anaerobic or relatively anaerobic bacteria. PAB are very small and take the form of spherical shape (cocci) under anaerobic conditions. However, in the presence of oxygen, they demonstrate pleomorphism in which club-shaped cells are observed; they can also take the form of letters V and Y. The optimal pH of PAB oscillates around 7.0 (range 4.5–8.0) in which they are characterized by their ability to produce propionic acid and vitamin B12, and they show increased growth rate even in the presence of 6.5% NaCl in their optimum pH. Most *Propionibacterium* spp. are mesophiles; however, they are resistant to much higher temperatures, and they can survive up to 20 s at 70 °C (certain strains withstand temperatures of up to 76 °C for 10 s). Their optimum temperature for growth is 30 °C. The following factors show an inhibitory effect on the *Propionibacterium* genus: high acidity, low/high temperature, high salt concentration, and water activity. Adaptation of PAB to one of the aforementioned stressors increase their resistance to other parameters (Kujawski et al. 1994; Boyaval et al. 1999; Koussémon et al. 2003; Leverrier et al. 2004; Benjelloun et al. 2007; Daly et al. 2010). PAB have significant growth preferences. In addition to the substances needed for their growth (source of carbon and nitrogen), they also need proper supplementation with microelements (iron, magnesium, cobalt, manganese, copper, amino acids, vitamins B7 and B5, and L-cysteine hydrochloride). Presence of aspartic acid in the environment favors the growth of PAB and increases their fermentation efficiency and carbon dioxide production (Fröhlich-Wyder et al. 2002). Their primary sources of carbon are saccharides (e.g., glucose, lactose, fructose, ribose, and galactose) and organic acids (lactic acid). They obtain nitrogen from peptides, amino acids, ammonium salts, and amines. They grow very slowly on the solid media and only under strictly anaerobic conditions, at a temperature of 30 °C and at optimum pH. Their growth lasts for up to 2 weeks when cultured on the lactate medium supplemented with glucose. Because of this, it is hard to identify and isolate them. Therefore, further studies are being conducted to develop molecular methods that might help in the detection of *Propionibacterium* in their habitat (Suomalainen et al. 2008). Colonies of PAB on the solid media may be of cream, orange, red, or brown color depending on the species; however, in the liquid media, they behave as a heavy fiber-like pellet.

*Propionibacterium* spp. have many valuable properties and from the technological point of view, the following are the most important: they can utilize lactose and lactates as carbon source, secrete intracellular peptidases and cell wall-associated proteases, synthesize compounds that have preservative properties (bacteriocins, propanoic acid, and acetic acid), they produce compounds that have aroma and taste (proline aminopeptidase-releases proline, which contributes to the sweet taste of cheese; they also have the capacity to convert free amino acids to aromatic compounds), and are capable of production of vitamin B12 (Hugenholtz et al. 2002). Some PAB possess generally recognized as safe (GRAS) and qualified presumption of safety (QPS) statuses, which means, if the bacteria have not been genetically modified then the live bacterial cells and their metabolites can be added to food/feed products.

## Industrial use of PAB

Bacteria from the *Propionibacterium* genus have found wide application in the cheese industry as a cheese microflora (together with lactic acid bacteria, which favors the environment for *Propionibacterium* strains), used in the production of hard rennet Swiss-type cheese (Swiss-Emmental cheese, Dutch-Leerdammer, French-Comté) and Polish medium-hard Swiss-type Dutch cheese (Tylżycki, Królewski). The role of these bacteria in cheese production is based on the fermentation of lactates to propionic and acetic acid, which gives a specific aroma to the final product; they also serve as natural preservatives (Thierry and Maillard 2002; Thierry et al. 2005). Starter cultures consisting of PAB and lactic acid bacteria (*Lactobacillus plantarum*, *Lactobacillus acidophilus*, *Penicillium jensenii*, and *Penicillium acidipropionici*) are being utilized in vegetable pickle production. Their combination increases the speed of the fermentation processand protects the final product against mold and rot, apart from the fact that pickles obtained by this method are vitamin B12 enriched and possess better taste and dietetic properties. Furthermore, *P. freudenreichii* subsp*. shermanii* induces apoptosis of the colon cancer cells, which is attributed to the production of propionate and acetate (Cousin et al. 2016). Moreover, studies have been published regarding the use of *P. freudenreichii* subsp*. shermanii* as a health-promoting additive in the Feta-type cheese in 2017. Colony-forming units (CFUs) of *Propionibacterium* in the ripening product increased up to 7 days of the process, and propionic acid concentration of 52.1 mM was achieved after 60 days. The obtained Feta-type cheese, in addition to imparting flavor, was characterized by health-promoting properties and also extended the expiration day (Angelopoulou et al. 2017). PAB strains are also used in feed production (Bioprofit™), which is the source of vitamin B12; they facilitate iron and calcium assimilation in animals and protect the final product against fungal infection. Some strains of PAB are used as probiotics for animal feeding. *P. freudenreichii* regulates intestinal microflora, stimulating growth of *Bifidobacterium* bacteria, and protects organism against growth of pathogenic microorganisms by generating bacteriocins. PAB have the ability to scavenge mycotoxins in the digestive tract. They stimulate the immune system and decrease mutagenic effects of fecal enzymes, and they also generate trehalose and vitamins B12, H, and folic acid. The addition of PAB to the feed results in its increased use which promotes the growth of young animals. Research is also being conducted on the use of live PAB as a substitute for preservatives with health-promoting effects in the milk product (e.g., cheeses and yogurt curd cheeses), fruit and vegetable products, and “bake-off” products (Langsrud et al. 1995; Mantere-Alhonen 1995; Piveteau 1999; Jan et al. 2002; Hojo et al. 2007; Zárate et al. 2002; Meile et al. 2008; Borawska et al. 2010, Ranadheera et al. 2010; Miks-Krajnik 2012).

## Biosynthesis of propionic acid

Propionic acid is an organic compound from the group of carboxylic acids (C_2_H_5_COOH). It is a colorless water-soluble liquid at room temperature with an unpleasant pungent odor. Propionic acid is primarily used as a preservative (E280); it inhibits the growth of yeast and molds. It is used as a preservative in prepacked sliced bread, rye bread, breads with reduced calories, and partially baked rolls, pita bread, and pastry products. It is also used as a preservative in animal feed. The maximum recommended concentration of propionic acid is 3000 mg/kg of the final product. Nearly 80% of the generated propionic acid is used in the food and the animal feed industry. It is an essential component of the cellulose fibers, herbicides, perfumes, and pharmaceuticals.

Three known pathways exist in the biosynthesis of propionic acid. One of them uses bacteria from *Clostridium propionicum*, *Bacteroides ruminicola*, and *Megasphaera elsdenii*. In these microorganisms, pyruvate obtained through glycolysis is firstly converted into lactate (in the presence of L-lactate dehydrogenase) and then lactoyl-CoA is generated as a result of propionate CoA-transferase activity, which is converted into acryloyl-CoA by dehydratase activity. Acryloyl-CoA is reduced to propionyl-CoA in the reaction catalyzed by acryloyl-CoA reductase at the final step. This pathway is inefficient as acryloyl-CoA is toxic to the bacterial cells. Inhibitory effects of acryloyl-CoA are directly correlated with the pH of the environment - minimal toxic concentration of acryloyl-CoA is proportional to the increase in pH. The presence of acryloyl-CoA in the environment causes increase in the molar ratio of acetic acid to propionic acid even up to 1:1 (theoretically it should be 1:2, such a profile of fermentation arises from the need to maintain a balanced redox state in the bacterial cells) (Erickson et al. 1979; Reichardt et al. 2014). According to a study (Kośmider et al. 2010a), the ratio of these compounds in the Wood-Werkman reaction might be 1:8. Thus, acryloyl-CoA may contribute to the increased production of acetic acid at the expense of propionic acid, thereby favoring the biosynthesis of acetic acid. Moreover, extraction of propionic acid by distillation is strongly inhibited by the presence of acetic acid. Low concentration of acetic acid results in improved efficiency and facilitates the procedure of obtaining pure propionic acid from the postculture liquid media. It is, therefore, important, from a technological point of view, that during the process of propionic acid production, it is important to maintain low efficiency of acetic acid biosynthesis (Barbirato et al. 1997).

Fermentation of propanediol is another known biosynthetic process used in the production of propionic acid. Some bacteria have the ability to synthesize 1,2-propanediol from deoxy sugars (e.g., fucose and rhamnose), dihydroxyacetone, and lactate. This pathway was identified in bacteria from *Salmonella enterica* and *Roseburia inulinivorans* and also from *Lactobacillus* genus. Lactaldehyde synthesized during biosynthesis is converted into 1,2-propanediol, and then into propanal, and finally into propionin in the presence of dehydrogenase. The efficiency of propionic acid production during the fermentation of propanediol depends on the source of carbon used. The primary fermentation products are acetic acid, formic acid, and lactic acid, when glucose is used as the carbon source. In such circumstances, propionic acid constitutes an insignificant percentage of the generated metabolites. Production of propionic acid increases when fucose or rhamnose is used as the carbon source; however, acetic acid remains a dominant product (Zang et al. 2010, Reichardt et al. 2014).

**Fig. 1:**
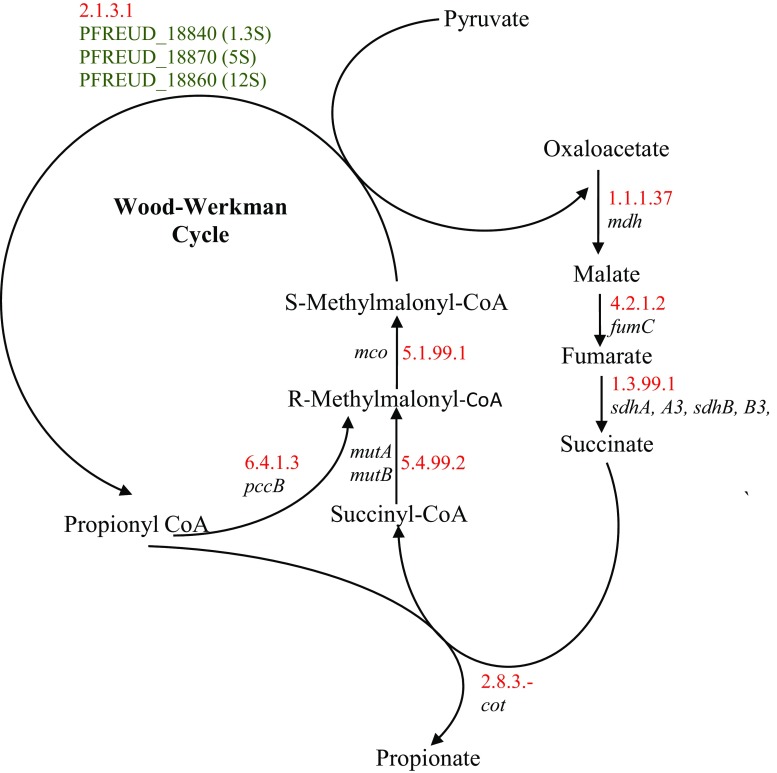
Route of propionic acid biosynthesis in *Propionibacterium* species (Falentin et al. 2010) *enzyme number (red), gene name (black), locus (green)

Wood-Werkman pathway is the third and the most important biosynthetic pathway of propionic acid production in which bacteria from *Propionibacterium* genus are utilized. By-products of this pathway are methylmalonyl-CoA, succinyl-CoA, and CO_2_. A key feature of the Wood-Werkman cycle in PAB is transcarboxylation reaction. The enzyme catalyzing this reaction is methylmalonyl-CoA carboxyltransferase, transferring carboxylic group from methylmalonyl-CoA into pyruvate with the generation of oxaloacetic acid and propionyl-CoA (Fig. 1). This enzyme is a biotin-dependent carboxytransferase (EC 2.1.3.1) and consists of three subunits (1.3S, 5S, and 12S) (Falentin et al. 2010).

Wood-Werkman pathway (Fig. 1) starts with the transformation of pyruvate generated during glycolysis into oxaloacetate in the presence of methylmalonyl-CoA carboxytransferase and biotin–CO_2_ complex. Then, oxaloacetate is reduced through malate and fumarate into succinate. In the next stage, succinate is acetylated by succinyl CoA synthetase into succinyl-CoA, which in cooperation with coenzyme B12 (cobalamin) and methylmalonyl-CoA mutase is transformed into methylmalonyl-CoA which next leads propionyl-CoA generation. CoA transferase releases CoA from propionyl-CoA, transforming it into propionate (Fig. 1). In addition to the bacteria from *Propionibacterium* genus, this pathway is also present in *Veilonella alcalescens* and *Selenomonas ruminantium* (Reichardt et al. 2014).

The Wood-Werkman pathway (Fig. 1) is the best from the perspective of propionic acid production. Compared to the other two pathways, in this pathway, the primary product of fermentation is propionic acid, which is very efficiently synthesized when compared to acetic acid and other by-products (Kośmider et al. 2010a; Wang et al. 2013). This pathway is also characterized by a wide range of possible sources of carbon to apply (rich *Propionibacterium* enzymatic system intensifies this effect). The advantage of this pathway is also the fact that none of the intermediate fermentation products are directly toxic to the cells that synthesize it. However, production of acids results in acidification of the environment, which inhibits further growth of the microorganisms, thereby decreasing the production of propionic acid (Zhang and Yang 2009). However, this can be easily rectified by neutralizing the production environment.

## Propionic acid production method

Propionic acid is currently synthesized via petrochemical processes (by the hydrocarboxylation of ethylene) that requires substantial financial expenditure and causes substantial damage to the environment. This results from the fact that chemical production of propionic acid is more economical than the microbial process utilizing PAB. The market price of the synthetic propionic acid costs $1000/ton, whereas the cost of 1 ton of acid produced via biotechnological processes with the participation of PAB may reach as much as $2000.

The global market of propionic acid production peaked at almost $935 MM in 2012. According to MarketsandMarkets, this figure should rise to at least $1.7 billion by 2018 (the vast majority involves the synthetic product). Developing countries in Africa and Asia may be responsive for such demands. Moreover, the increase in demand for propionic acid from microbial origin is assigned to the growing needs of communities in North America and European countries, where the growing interest in ecological products is observed. This increase in demand for propionic acid is related to the introduction of the products with “clean labels” with no artificial additives on the market (Baumann and Westermann 2016). Considering the scarcity of resources and serious environmental damages caused due to the chemical production of propionic acid, as well as due to the rise in demand for natural and ecological food products, there is an increasing demand in the microbial production of propionic acid with the usage of waste products generated from various industries. This should measurably reduce the cost as well as should improve the environmental status. To this end, the search for the new metabolic pathways is necessary to intensify the biosynthesis of metabolites in PAB (Chen et al. 2012; Wang et al. 2013; Guan et al. 2015c).

Bacteria from *P. freudenreichii*, *P. jensenii*, *P. thoenii*, and *P. acidipropionici* species seem to be the most appropriate for the biotechnological production of propionic acid. Due to their wide variety of enzymatic systems, they can utilize carbon from various sources, pure and from waste products (Boyaval and Corre 1987; Carrondo et al. 1988; Hsu and Yang 1991; Lewis and Yang 1992; Quesada-Chanto et al. 1994; Barbirato et al. 1997; Ramsay et al. 1998; Himmi et al. 2000; Huang et al. 2002; Yazdani and Gonzales 2007; Zhu et al. 2010; Feng et al. 2011; Zhu et al. 2012; Piwowarek et al. 2016).

Utilizing waste products that are generated from the technological processes is one of the significant problems of manufacturing companies and environmentalists. Adequate waste management has many benefits, including limited environmental pollution and clean-up costs, improving hygiene, or the possibility of acquiring low-cost new products. Therefore, researchers are constantly seeking innovative solutions to manage industrial waste, especially in biotechnology. Waste products are a frequent reservoir of the biologically active compounds (can be good source of carbon, proteins, pectin, fiber, vitamins, and organic acids). Therefore, it is advisable to consider waste products as reservoirs of valuable materials that can be further processed. This will reduce the cost of the media enrichment, which will result in cheaper products. Biotechnological utilization of bacteria can result in the reduction of environmental pollution not only through the disposal of waste, but also through their transformation into useful and valuable industrial compounds, such as propionic acid, which is currently being sourced via chemical production (Cybulska et al. 2013, Piwowarek and Lipińska 2015).

Batch and semi-continuous culture methods are frequently utilized in the production of propionic acid (Barbirato et al. 1997; Zhu et al. 2010). Propionic acid production with use of PAB can have feedback inhibition by the extensive accumulation of by-products, primarily acetic acid (it lowers the pH, thus inhibits bacterial growth) (Suwannakham and Yang 2005). Extractive fermentation was performed to reduce the effect of the generated acids on the production of propionate (Jin and Yang 1998; Zhu et al. 2012). Fermentation process performed under these conditions also has disadvantages, such as reduced effectiveness of the process resulted from the presence of the extraction compound in the culture which increased osmotic pressure (Kourkoutas et al. 2005; Meynial-Salles et al. 2008). Suwannakham and Yang (2005) performed immobilized culture of *P. acidipropionici* ATCC 4875 inside the bioreactor, in order to reduce the effect of the acids on the metabolic activity of bacteria. Immobilized cells produced a much higher amount of propionic acid (71.8 g/L), which indicates that the bacterial cells showed increased resistance toward generated acids. This method produced 20–59% more propionate, 17% less acetic acid, and 50% less succinate compared to free cells. Sugar cane stalks were used by Chen et al. (2012) for the immobilization of *P. freudenreichii* CCTCC M207015. The highest concentration of propionic acid (136.23 g/L) was obtained via continuous fermentation, which increased by 21.07% compared to the free cells. In each case (Suwannakham and Yang 2005; Chen et al. 2012), were observed morphological changes in the immobilized cells, such as threefold increase in length, decrease in the diameter, and increase in surface area, which most likely resulted in more effective transportation of the compounds and metabolites across the cell membranes. This, in turn, increased the production of propionic acid by bacteria.

## Research for improved biosynthesis of propionic acid by using genetic engineering

Propionic acid is primarily produced via petrochemical processes; however, there is increasing interest in obtaining this compound via fermentation of the renewable biomass. Propionic acid biosynthesis with the use of bacteria from the *Propionibacterium* genus is unfortunately characterized by low efficiency of the process from an industrial point of view. Thus, attempts to intensify the fermentation process via genetic engineering tools are on. Few strategies were performed successfully. For example, Suwannakham et al. (2006) improved propionic acid production from *P. acidipropionici* ATCC 4875 by deleting *ack* gene that encodes acetate kinase from its genome. This resulted in the inhibition of acetic acid production and thereby increasing the production of propionic acid. *P. acidipropionici* mutant (deprived of gene coding acetate kinase) was used by Zhang and Yang (2009) during their study on tolerance of PAB to the acidic condition by immobilizing the bacteria on a fibrous-bed reactor. After about 3 months of adaptation of the mutant, the concentration of propionic acid in the fermentation broth reached 100 g/L, which was substantially higher than the concentration of the metabolite with the use of the wild-type strain (71 g/L). The immobilized mutant was characterized by decreased susceptibility to the acids produced resulting from the increased activity and expression of gene coding H^+^-ATPase, which is related to the proton pumping and cell’s ability to control its intracellular pH gradient. Overexpression of genes encoding glycerol dehydrogenase (GDH), malate dehydrogenase (MDH), and fumarate hydratase (FUM) via genetic engineering improved the production of propionic acid by Liu et al. (2015). Activity of these enzymes in the modified strain was found to be from 2.91 to 8.12 higher than that of wild-type *P. jensenii*, whereas the level of transcription increased from 2.85 to 8.07. Coexpression of GDH and MDH increased the production of propionic acid from 26 to 39 g/L.

Wang et al. (2015a) analyzed the effects of overexpression of three biotin-dependent enzymes, namely, pyruvate carboxylase (PYC), methylmalonyl-CoA-decarboxylase (MMD), and methylmalonyl-CoA transcarboxylase (MMC) that are responsible for the carbon flux in the Wood-Werkman cycle. Mutants with overexpression of MMC and MMD were characterized by increased synthesis of propionic acid and reduced production of acetic and succinic acids compared to the wild-type strain. However, the growth of the mutants overexpressing PYC was slower, produced more succinate, and had 12% lower efficiency of the propionate biosynthesis. Wang et al. (2015b) inspected the effect of overexpression of native propionyl-CoA/succinate CoA transferase (CoAT) in the cells of *P. shermanii* on the production of propionic acid from glucose and glycerol. The mutated strain produced more propionic acid and was characterized by 10% increased efficiency. Overexpression of CoAT might have resulted in directing carbon content into propionic acid biosynthesis, thereby increasing efficiency. In other studies, Ammar et al. (2014) cloned gene encoding phosphoenolpyruvate carboxylase (PPC) from *Escherichia coli* into *P. freudenreichii*. PPC catalyzes conversion of oxaloacetate into phosphoenolpyruvate in the presence of CO_2_. Overexpression of PPC in *P. freudenreichii* significantly changed fermentation of propionic acid. PPC-overexpressing mutants more effectively utilized glycerol and produced propionate faster than wild-type. This can be attributed to more efficient binding of CO_2_ and changes in the dicarboxylic acid pathway.

Bacteria from *P. freudenreichii* subsp. is cannot utilize xylose, a sugar which is abundant in woody biomass. Wei et al. (2016) identified three genes in the catabolic pathway of xylose in *P. acidipropionici*: xylose isomerase (*xylA*), xylose transporter (*xylT*), and xylulokinase (*xylB*). Overexpression of these genes in *P. freudenreic*hii subsp*. shermanii* cells was performed using expression vector pKHEM01, enabling the mutant with effective utilization of xylose, even in the presence of glucose. The generated mutant was characterized by similar fermentation kinetics of glucose, xylose, and glucose/xylose mix. Constructed strain of *P. freudenreic*hii subsp*. shermanii* may thus represent a potential alternative for the industrial manufacturing of propionic acid and other high value-added products from ligno-cellulosic biomass (Liu et al. 2012).

Biosynthesis of propionic acid is controlled in a feedback mechanism in bacteria from the *Propionibacterium* genus. Increased bacterial resistance against acids is the most effective strategy to increase the biomass of PAB and subsequently propionic acid synthesis (Guan et al. 2014). To achieve this, Guan et al. (2012) used adaptive evolution and genome shuffling. A significant role of arginine deiminase (EC 3.5.3.6) and glutamate decarboxylase (EC 4.1.1.15) in the bacterial tolerance against acids in *P. acidipropionici* cells (Guan et al. 2013; Zhang and Yang, 2009) was also determined. Guan et al. (2015b) tried to improve resistance of *P. jensenii* ATCC 4868 against effect of acids through overexpression of five genes: *Arca*, *ARCC*, *gadB*, *GDH*, and *ybaS*, encoding, among others, glutamate dehydrogenase and arginine deiminase. The most positive effect on the bacterial resistance against propionic acid and efficiency of its production resulted from the overexpression of *gadB* (coding glutamate decarboxylase). Resistance of *P. jensenii* against acid increased more than 10 times (compared to the wild-type strain), and the efficiency reached a total amount of 5.92 g/g glycerol (increased by 23.8%). Their results have confirmed that the expression of genes via genetic engineering resulted in change in the amino acid pool and an additional expression of other genes, which may have contributed to the increased biosynthesis of propionic acid. This is an effective strategy to increase the production of propionate with use of bacteria from the *Propionibacterium* genus. This strategy can be useful in the production of other organic acids. Current scientific knowledge of the functioning of the resistance against acid in the cells of *Propionibacterium* remained at the microenvironmental level. Thus, further testing is required to understand these mechanisms. Methods from systems biology could be useful in this regard. Technologies comparing genomics and transcriptomics may be used to obtain bacterial strains resistant against acids at the DNA level, whereas proteomics and metabolomics may be used to identify key proteins and metabolites, as well as pathways responsible for a particular feature. Systems biology, which involves introduction of features from one organism into another, can also be used to improve resistance of *Propionibacterium* to low pH. To achieve this, elements responsible for resistance toward acids that are identified in other bacteria, will be introduce into *Propionibacterium* (Guan et al. 2015a, 2015b). For example, Lu et al. (2013) identified a new system of acid resistance in *E. coli*, in the reaction where L-glutamine is converted into L-glutamic acid with release of ammonia. Elements responsible for this change in *E. coli* may become applicable in *Propionibacterium* to increase resistance toward acidic conditions. The limitation factor of metabolic engineering of bacteria from the *Propionibacterium* genus is the restriction modification (RM) system, which decreases the cell’s transformation capabilities. This is a major setback to the genetic manipulations (Van Luijk et al. 2002). These systems coordinate activity of restriction and modifying enzymes to distinguish foreign DNA from host DNA, thereby protecting the cells against introduction of the foreign genetic material.

Application of systems biology and methods of synthetic biology may solve the problems and provide new data and possibilities, which extend the scope of *Propionibacterium* application. A true revolution in terms of breaking limitations of the RM system in PAB cells may be a method of DNA modification, based on the Cas9–CRISPR–Cas9 protein (clustered regularly interspaced short palindromic repeats (CRISPR) associated). This system utilizes the elements of acquired immunity in bacteria and archaea in response to phage infection and genetic transformation with new genetic material. Microorganisms include fragments of foreign DNA into their CRISPR loci in the genome, allowing future fast recognition and eradicating infection. Cas9 can be used to introduce stable changes in the genome (knock-out and knock-in), among others, in the genetic modification processes and activation or silencing of selected genes (Jinek et al. 2012; Wiedenheft et al. 2012; Cong et al. 2013; Jiang et al. 2013; Ran et al. 2013; Ousterout et al. 2014).

## Biosynthesis of vitamin B12

Vitamin B12 is a general term used for the compounds from the cobalamin group. This includes four basic chemical forms: cyanocobalamin, in which cobalt is substituted by the CN- group, hydroxocobalamin with the OH- group, methylcobalamin with the CH- group; and deoxyadenosylcobalamin containing 5-deoxyadenosyl moiety. Vitamin B12 belongs to corrin compounds (cobalamins). Cobalamin molecule consists of four pyrrole subunits (A–D) connected with each other in alpha position, thereby establishing a macrocyclic structure. First subunit is conjugated with fourth (A and D) directly through the Cα-Cα chemical bond. This structure binds with centrally positioned carbon atom with two coordinated ligands (upper and lower), for example, cyanidic group, adenosine and 5,6-dimethylbenzimidazole (DMBI). DMBI is responsible for the therapeutic properties of vitamin B12 in humans. Presence of another substituent such as adenine in DMBI position forms the so-called pseudovitamin B12, which is active only in the bacterial cells (Raux et al. 1999; Beck 2001; Martens et al. 2002). Vitamin B12 can be synthesized only by the bacterial cells and the archaebacteria and, among others, by the soil microorganisms, by the microflora in the digestive tracts of humans and animals, natural fertilizers, and wastewater. Milk, cheese, eggs, meat of the ruminants, poultry, fish, crustaceans, and meat offal are the sources of vitamin B12 in human diet (Rodionov et al. 2003; Ortigues-Marty et al. 2006; Smith et al. 2007). In humans, the recommended cobalamin intake depends on the age and physiological status of the person. The optimal daily dosage for women and men (aged ≥ 14 years) is 2.4 μg, whereas the daily dosage for pregnant and breast-feeding women is from 2.6 to 2.8 μg. Vitamin B12 is essential in erythropoiesis (red blood cell formation in bone marrow) and in many other functions in the human organism (Fenech 2001; Knasmüller and Verhagen 2002; Paoloni-Giacombino et al. 2003; Kolling et al. 2004; Luggen 2006; Smith et al. 2007). The severe effects of vitamin B12 deficiency are anemia, atherosclerosis, heart diseases, neurological diseases (paralysis of limbs, ataxia, and lethargy), increased susceptibility of the deoxyribonucleic acid (DNA) to damages, methylation changes, and other (Aleman et al. 2001; Fenech 2001; Dharmarajan et al. 2003; Figlin et al. 2003; Luggen 2006).

Cobalamin is synthesized via two mechanisms: aerobic (with the participation of *cob* genes present in bacteria from *Pseudomonas* genus) and anaerobic (with the participation of *cbi* genes present in bacteria from *Bacillus* and *Salmonella* genus). Bacteria from the *Propionibacterium* genus need both anaerobic and aerobic conditions (genome of these bacteria has genes with prefix *cbi* and *cob*) to effectively produce vitamin B12 (Scott 1994; Raux et al. 1996; Raux et al. 1998; Martens et al. 2002; Falentin et al. 2010).

**Fig. 2:**
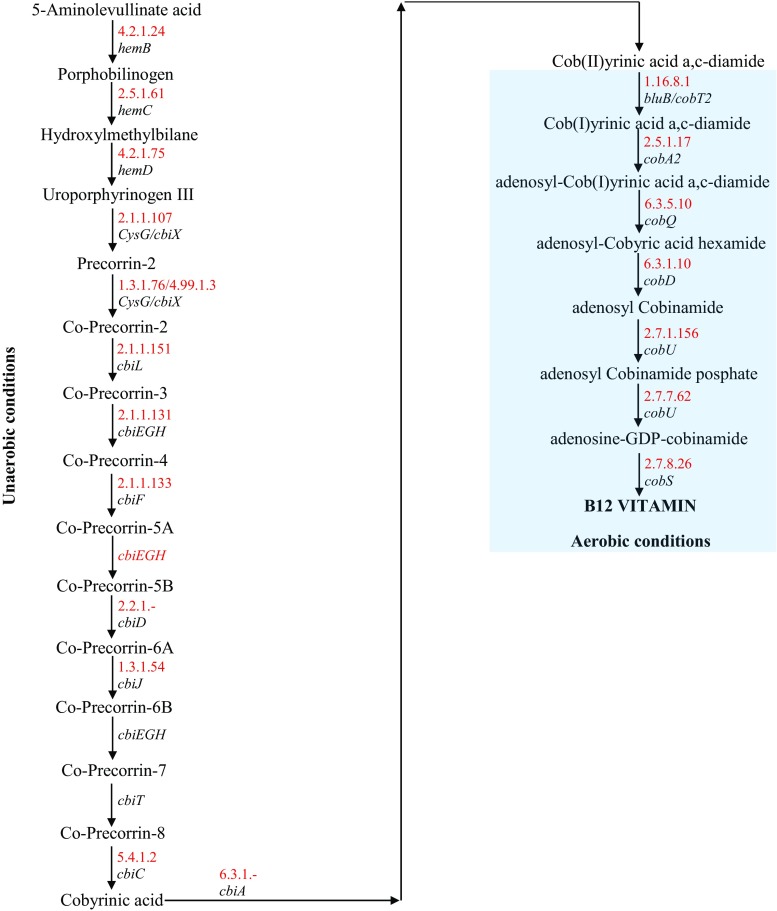
Vitamin B12 biosynthesis by the bacteria from *Propionibacterium* genus (Falentin et al. 2010) *enzyme number (red), gene name (black)

Biosynthesis of all tetrapyrrolic derivatives in plants, archaea, and most bacteria starts with glutamate C5 backbone. The first step is the addition of glutamate to tRNA by the action of glutamyl-tRNA^Glu^ synthetase. This reaction requires hydrolysis of one molecule of ATP into AMP and PPi. Next, tRNA^Glu^ is reduced into glutamate-1-semialdehyde, and this reaction is catalyzed by glutamyl-tRNA reductase. The formed glutamate-1-semialdehyde is then converted into 5-aminolevulinic acid (ALA) by glutamate-1-semialdehyde aminotransferase, first general precursor to any known tetrapyrroles (intrmolecular transfer of the amino group between C-2 and C-1 of semialdehyde). Adenosylcobalamin is produced from uroporphyrinogen III, which is derived from eight molecules of 5-aminolevulinic acid. The first steps in the production of vitamin B12 are performed under anaerobic conditions, and they are catalyzed by the enzymes coded by the genes with *cbi* prefix (Fig. 2). Synthesis of vitamin B12 starts with dimerization of 5-aminolevulinic acid molecules, resulting in the generation of porphobilinogen (PBG). The next step is polymerization of four PBG molecules, resulting in the formation of pre-uroporphyrinogen. This compound subsequently undergoes inversion and cyclization generating biologically active uroporphyrinogen III—a precursor of corrin structure. Three enzymes participate in the aforementioned reactions: 5-aminolevulinic acid dehydratase (*hemB*), porphobilinogen deaminase (*hemC*), and uroporphyrinogen III synthase (*hemD*). Action of uroporphyrinogen-III C-methyltransferase results in the methylation of this compound at C-2 and C-7 positions, causing the prototype version of the ring (*precorrin-2)* formation. Directly after being generated, the prototype of the corrin ring binds cobalt. This reaction is catalyzed by ATP-independent chelatase. Carbon C-20 is removed after the binding of cobalt and oxidation in the form of acetaldehyde, which is related to the presence of cobalt that can have different oxidation states (from + 1 to + 3). In the next nine reactions that are catalyzed by different types of enzymes (Fig. 2), involving methylation of carbon at the appropriate positions, corrin ring is converted into cobinamide. This is achieved by the conjugation of aminopropanol with propionic acid moiety attached to the side chain of the D ring. The following reactions require the presence of oxygen as they are catalyzed by the enzymes coded by the genes with *cob* prefix (Fig. 2). Consequently, the lower ligand is created, and upper and lower ligands are attached to cobinamide. Formation of nucleotide present under the macrocyclic ring results from the transfer of phosphoribosyl moiety of nicotinamide mononucleotide onto DMBI to produce α-rybazol. Then, α-rybazol is attached in the presence of GDP to adenosylcobamamide, which releases GMP. All the aforementioned transformations lead to the generation of the complete form of adenosylcobalamin (Fig. 2) (Lamm et al. 1982; Blanche et al. 1989; Warren et al. 1990; Louie et al. 1992; Jordan 1994; Sattler et al. 1995; Warren et al. 1998; Roessner et al. 2002; Warren et al. 2002; Piao et al. 2004a, 2004b; Falentin et al. 2010; Kośmider and Czaczyk 2010b; Khan Mazharuddin et al. 2011, Chamlagain 2016).

Active vitamin B12 differs from pseudovitamin by the presence of DMBI at lower ligand position of the macrocyclic ring. According to Deptula et al. 2015, the *P. freudenreichii* genome has fusion enzyme BluB/CobT2 implicated in production of the active form of vitamin B12. Understanding the mechanisms affecting the synthesis of different forms of cobalamin is important in the context of selection of strains and increasing the production of vitamin B12. Thirty genes are implicated in the biosynthesis of vitamin B12 in *P. freudenreichii* (Roth et al. 1993). The most important from the industrial point of view are the final steps of the pathway (production, activation, and attachment of lower ligand) that determine the generation of therapeutically active vitamin. Enzymatic complex BluB/CobT2 is the key in the biosynthesis of active vitamin B12 (Fig. 2). DMBI is generated via reduction of FMN catalyzed by BluB enzyme in anaerobic bacteria. The generated DMBI is then activated by CobT2 enzyme (CobT2 is responsible for selective introduction of DMBI into cobinamide), resulting in the production of α-ribazole phosphate, which is then attached to the macrocyclic ring creating complete molecule of active form of cobalamin. Between seven analyzed homologs of CobT2 derived from different microorganisms, all of them were characterized by affinity to DMBI and lack of the ability to utilize other substrates, which prevents the biosynthesis of inactive compounds. Low level of pseudovitamin B12 production, which is caused due to the lack of the ability of enzymatic complex to utilize, for example, adenine, suggests that *P. freudenreichii* bacteria prefer the active form of cobalamin as a cofactor of enzymatic processes (Falentin et al. 2010; Deptula et al. 2015, Chamlagain 2016).

All *Propionibacterium* strains capable of active production of vitamin B12 may produce it only in the presence of oxygen. It is related to the oxygen-dependency of DMBI ligand. Therefore, vitamin B12 production with the use of *Propionibacterium* strains can be divided into two phases: in the first phase, bacterial cells should be cultured under anaerobic conditions for the first few days to generate the precursor of vitamin B12, that is, cobinamide (intermediate which lacks DMBI group). In the second phase, biosynthesis of active cobalamin ends by delicate airing of the culture for the next few days, when lower ligand is synthesized and is conjugated with previously generated cobinamide (Fig. 2). It is also important to maintain neutral pH and proper temperature (respectively: pH 7.0 and temp. 30 °C) (Miyano et al. 2000; Leman 2007) of the production environment for efficient production. It is, therefore, necessary to remove propionic and acetic acid generated during the fermentation, for example, via alkalization of the media, “cross-flow” filtration (Hatanaka et al. 1998), fermentation with purification on the activated charcoal-packed column (Nakano et al. 1996), extraction fermentation (Zhang et al. 1993), electrodialysis (Lewis and Yang 1992), or by immobilizing bacterial cells (Yang and Huang 1995; Czaczyk et al. 1997a; Czaczyk et al. 1997b). In order to maintain effective production, culture media should be supplemented with important compounds or precursors during the biosynthesis of vitamin B12, such as cobalt ions, DMBI, glycine, threonine, 5-aminolevulinic acid, betaine (present in beet molasses), and choline, regardless of the production strains used (Roman et al. 2001).

## Industrial production of vitamin B12

Due to the complexity (approximately 70 stages) and high costs of chemical synthesis of cobalamin, its industrial production is solely based on fermentation processes with microorganisms, typically *P. denitrificans* (Blanche et al. 1995; Blanche et al. 1998; Piao et al. 2004a, 2004b). In recent years, enrichment of food products with vitamin B12 via in situ fermentation has increased. In this context, it is noteworthy that *P. freudenreichii* is the only microorganism used with GRAS status, which can synthesize the active form of vitamin B12, which makes it a unique solution in the commercial production of the vitamin, food, and feed microbiological supplementation with it. Moreover, *P. freudenreichii* bacteria produce therapeutically active vitamin B12 with the concomitant minor production of inactive analog. Known are other organisms producing B12 vitamin, and which are classified as GRAS species, yet due to different causes they are less attractive for the industry than the PAB. For instance, *Lactobacillus reuteri* bacteria are unable to include ligands other than adenine in the lower part of the ring; they synthesize vitamin B12 that is inactive for humans (Santos et al. 2007; Crofts et al. 2013).

There are two ways to synthesize vitamin B12 by *Propionibacterium*: in the first method, bacterial cultures are used, which are responsible for the enrichment of certain fermented food products with vitamin B12 (Van Wyk et al. 2011). Second, the chemical pathway of vitamin B12 production, which is labor intensive and rather expensive, is replaced with microbial synthesis.

The Aventis Company (vitamin B12 production leader) prefers to use *P. denitrificans* (which lacks the status) for the industrial production of cobalamin. Vitamin B12 biosynthesis by *P. denitrificans*, contrary to *P. freudenreichii*, takes place in pure aerobic conditions. The combination of genetic engineering with concomitant mutagenization procedures enabled scientists from the Rhône-Poulenc-Rorer to obtain a *P. denitrificans* strain producing vitamin B12 on the level of 300 mg/L (Blanche et al. 1995). Blanche et al. (1989) describe the amplification of eight genes of *cobF–cobM* operon, which resulted in 30% increase in the production of cobalamin. A further enhancement of the biosynthesis of the metabolite (by 20%) was possible by increasing the copies of *cobA* and *cobE* genes. The genetically modified *P. denitrificans* strain enables production of vitamin B12 covering 80% of its global demand (Martens et al. 2002).

To improve the efficiency of cobalamin production, *P. freudenreichii* strain was genetically manipulated. Piao et al. (2004a, 2004b) enabled 2.2-fold increase in the biosynthesis of cobalamin, yet their efficiency was found to be low when compared with *P. denitrificans*. They expressed the genes participating in the cobalamin biosynthesis (*hem*, *cob*, and *cbi*). The recombined clone of *P. freudenreichii*, possessing the pPK705 expression vector with *cobA*, *cbiLF*, or *cbiEGH* insert produced respectively 1.7-, 1.9-, and 1.5-fold more cobalamin than the wild strain. Scientists (Piao et al. 2004a, 2004b) also introduced the *hemA* gene into the expression vector isolated from *Rhodobacter sphaeroides* cells and endogenic genes *hemB* and *cobA*, thus obtaining 2.2-fold more vitamin B12 than when using *P. freudenreichii* containing the pPK705 vector.

The use of genetically modified microorganisms in the production of metabolites for human health prophylaxis is a matter of considerable controversy. Therefore, numerous studies have been conducted to optimize vitamin B12 production using microorganisms that are not subjected to genetic modifications. These studies are devoted to the search of strains characterized by naturally high efficiencies of vitamin B12 biosynthesis or efficiency of the culture method (Table 1). Moreover, attempts have been made to improve the efficiency of cobalamin biosynthesis by optimizing the composition of fermentation media by selecting the most appropriate carbon sources (Quesada-Chanto et al. 1994), addition of micronutrients (Trojanowska and Czaczyk 1996) and cobalt ions (Seidametova et al. 2004), and use of different factors at the same time (Chiliveri et al. 2010). A considerable enhancement in the production of vitamin B12 was obtained by enriching the production media with precursors (Marwaha et al. 1983; Murooka et al. 2005) and analogs of vitamin B12 (Thirupathaiah et al. 2012).

**Table 1:** Production of vitamin B12 by selected strains from the *Propionibacterium* genus

Strains	Carbon source	Production of vitamin B12	References
*P. acidipropionici* DSM 8250	Beet molasses	34.8 mg/L	Quesada-Chanto et al. 1994
*P. acidipropionici* DSM 8250	Reed molasses	28.8 mg/L	Quesada-Chanto et al. 1994
*P. freudenreichii* subsp*.* shermani*i OLP-5*	Glucose	31.67 mg/L	Thirupathaiah et al. 2012
*Propionibacterium freudenreichii* NCIB 1081	Glucose	4.3 mg/L	Czaczyk et al. 1997a
*Propionibacterium shermanii* FRDC Pr1	Fermentation liquors (lactic fermentation)	1.8 μg/L	Gardner and Champagne 2005
*Propionibacterium shermanii* PZ-3	Glucose	52 mg/L	Hatanaka et al. 1998
*P. freudenreichii* subsp. *shermanii* DSM 20270	Waste from tofu production	10 mg/L	Yu et al. 2015
*Propionibacterium freudenreichii* CICC 10019	Glucose, corn extract	42.6 mg/L	Wang et al. 2012

Wang and Yang (2013), by cofermenting glucose and glycerol by their gradual addition, were able to obtain relatively high quantities of vitamin B12 and propionic acid using *P. freudenreichii* subsp*. shermanii* (0.72 mg/g and 0.71 g/g, respectively). The use of both carbon sources separately led to inferior results. Wang et al. (2013) demonstrated that an integrated fermentation system may provide an efficient method for a cost-effective and ecological production of propionic acid and vitamin B12.

A key factor limiting the production of active vitamin B12 by *Propionibacterium* bacteria is the biosynthesis of lower ligand of the metabolite, that is, DMBI. Chamlagain et al. (2016) tested the influence of DMBI precursors (riboflavin (RF) and nicotinamide (NAM)) and DMBI on the production of vitamin B12 by *P. freudenreichii* and *P. acidipropionici* in whey-based medium. The concomitant supplementation of media with RF (40 mM) and NAM (27 mM) increased the production of vitamin B12 fourfold in comparison to control cultures. For many strains, the efficiency of the vitamin production was comparable or higher than that obtained through the addition of DMBI (100 mM). Chamlagain et al. (2016) demonstrated that the availability of RF and NAM increases the production of active vitamin B12 by *P. freudenreichii* depending on the strain.

Wang P. et al. (2015) tested the influence of propionic acid and DMBI on the production of vitamin B12 combined with purification in an absorption column using a bioreactor. Consequently, the authors determined that maintaining an initial and subsequent propionic acid concentration of respectively 10–20 and 20–30 g/L can increase cobalamin production in an efficient manner. Their method that involves controlling the amount of propionic acid and DMBI resulted in the efficiency of vitamin B12 (58.8 mg/L).

## Biosynthesis and role of trehalose in *Propionibacterium*

Natural trehalose consists of two glucose molecules bound by an α,α-1,1′-*O*-glycosidic bond. Due to the low energy of the glycosidic bond, it is the most thermodynamically and kinetically stable disaccharide found in the nature. Because D-glucopyranosyl units are bound by the participation of anomeric carbon atoms, trehalose does not have reducing properties.

Trehalose is widely distributed in living cells, for example, in yeasts, fungi, plants, and microorganisms (nematodes, crustaceans, and insects) it was also identified in numerous bacterial cells, including *Propionibacterium*. The presence of trehalose in PAB has already been described 50 years ago (Stjernholm 1958), yet metabolism of the trehalose disaccharide in these bacterial cells has only recently been understood.

Trehalose disaccharide plays different roles in different organisms. They perform a protective role in response to stress induced by, among others, high or low temperature, dehydration, or changes in osmotic pressure. *P. freudenreichii* bacteria are capable of accumulating high levels of trehalose, particularly under stress conditions. Five metabolic pathways of trehalose are known: OtsAB, TreS, TreYZ, TreP, and TreT (Avonce et al. 2006). First two pathways are utilized by *Propionibacterium* bacteria. Following the reports of Cardoso et al. (2007), synthesis of trehalose by *P. freudenreichii* takes place via the OtsAB pathway, whereas TreS is responsible for its catabolism.

**Fig. 3:**
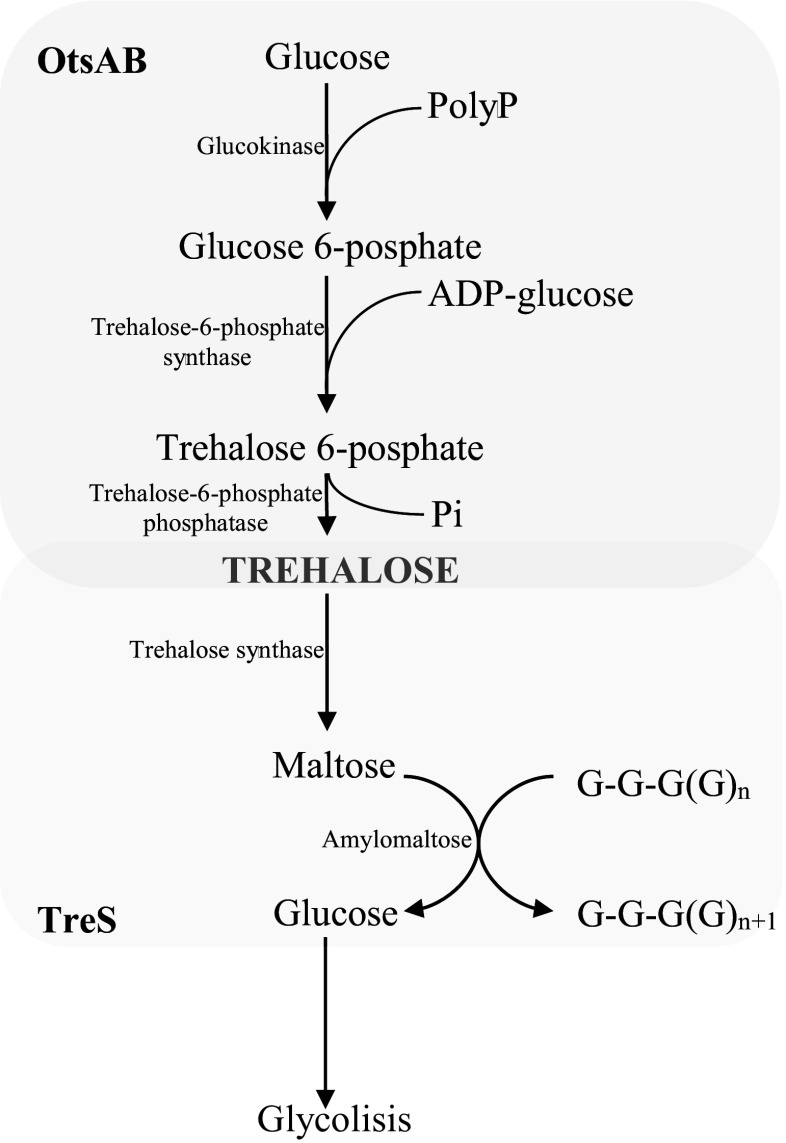
Scheme of metabolism of trehalose in *P. freudenreichii* (Cardoso et al. 2007; Ruhal et al. 2013)

The OtsAB pathway includes two enzymatic reactions that are catalyzed by trehalose-6-phosphate synthase and trehalose phosphatase: the first enzyme catalyzes the transformation of UDP-glucose by glucose-6-phosphate to trehalose-6-phosphate, which is then hydrolyzed by phosphatase to trehalose (Fig. 3). In the bacteria capable of using starch, glycogen, or maltodextrin as the carbon source, a different trehalose biosynthesis pathway takes place, which is catalyzed by two enzymes: maltooligosyl trehalose synthase (TreY) and maltooligosyl trehalose trehalohydrolase (TreZ) (De Smet et al. 2000; Cardoso et al. 2007). TreY impacts the α-1,4-glycosidic bond on the reducing end of maltodextrin, thereby transforming the bond into α,α-1,1-glycosidic bond, resulting in the formation of maltooligosyl trehalose. Subsequently, TreZ catalyzes the hydrolysis of the second α-1,4-glycosidic bond of maltooligosyl trehalose, leading to the release of trehalose (Elbein et al. 2003). In certain bacteria (e.g., *Pimelobacter* sp*.*), trehalose synthase (TreS) was detected that catalyzes the intracellular regrouping of maltose to trehalose. TreS isomerizes α-1,4-glycosidic bond of maltose to α,α-1,1-glycosidic bond forming trehalose (Elbein et al. 2003). Subsequently, the enzyme trehalose phosphorylase (TreP) participates in the biosynthetic pathway of trehalose disaccharide, taking place in certain fungi. TreP catalyzes hydrolytic release of trehalose from trehalose-6-phosphate that was formed earlier from glucose and glucose-1-phosphate. The last known trehalose biosynthetic pathway was determined in hyperthermophilic cells of *Thermococcus litoralis* archaeon. In these cells, trehalose glycosyltransferase synthase (TreT) is involved, which catalyzes the reversible biosynthesis of trehalose from ADP-glucose and glucose (Qu et al. 2004; Ryu et al. 2005).

Trehalose-6-phosphate synthase has been identified in numerous bacteria, yeast, mold, plant, and insect species. Depending on the species, a variable specificity of OtsA toward the source of glucose exists. In case of *P. freudenreichii*, as long as OtsA proteins are under stress conditions, they exhibit affinity to ADP-glucose. In case of raw *P. freudenreichii* extracts, OtsA solely utilizes ADP-glucose, whereas pure recombined OtsA, apart from ADP-glucose, also utilizes UDP-, GDP-, and TDP-glucose. This suggests that the regulatory protein, determining the specificity of OtsA toward ADP-glucose, is the second protein of the OtsB pathway (Fig. 3) (Cardoso et al. 2007).

In response to osmotic stress or decreasing pH of the environment, *P. freudnereichii* shows an increase in the level of trehalose-6-phosphate synthase of the OtsAB pathway, whereas the level of enzymes of the TreS pathway remains unaltered or decrease slightly. This confirms that under stress conditions for *Propionibacterium* species —trehalose is synthesized only via the OtsAB pathway. The absence of common degradation pathways of trehalose in the extract from *P. freudnereichii* suggests that the TreS pathway is responsible for the activity in these cells, resulting in the decomposition of trehalose to maltose (Fig. 3). High activity of amylomaltase in the extract from *P. freudnereichii* indicates that from the maltose reducing end of maltooligosaccharides, obtained through TreS glucose is released, subsequently catabolyzed in the glycolysis pathway (Cardoso et al. 2007; Othake and Wang 2010).

The physiological role of numerous trehalose pathways was clearly determined only in several bacterial species (De Smet et al. 2000; Wolf et al. 2003; Makihara et al. 2005). Strong evidences in the degradation of trehalose via the TreS pathway were observed in *Sphaeroides rhodobacter* mutants (Makihara et al. 2005). A similar TreS pathway has also been proposed for *Corynebacterium glutamicum* cells. The course of reaction catalyzed by TreS in these bacteria is directed both toward the synthesis and degradation of trehalose. Trehalose synthesis takes place solely when maltose is the only carbon source available in the environment (Wolf et al. 2003), in the remaining cases, TreS is responsible for the decomposition of trehalose to maltose.

Trehalose is accumulated in the cells of *P. freudenreichii* in response to osmotic, oxidative, acidic, and thermal stress. It clearly suggests a protective function of this sugar against the aforementioned factors. During stress conditions, such as elevated osmolarity, trehalose is responsible for maintaining the cell’s elasticity. The role of trehalose as an oxygen radical scavenger has been demonstrated in *Sacchraomyces cerevisiae* (Benaroudj et al. 2001; Chi et al. 2001). Accumulation of trehalose in reaction to the effect of elevated temperature has been explained in a range of mesophilic organisms, such as yeasts and bacteria (*E. coli* and *S. enterica)* (Strom 1993; de Virgilio et al. 1994; Cánovas et al. 2001). Genes of trehalose synthase have also been determined in the genome of *Picrophilus torridus* bacteria, which has the optimum growth pH of 0.7. It is most likely for this microorganism that trehalose is involved in the stabilization of the cell membrane, which has to withstand strong pH gradient in the environment.

Maltose, sucrose, and glucose are the carbon sources commonly used in research for the production of trehalose. However, they have been gradually replaced with waste from different branches of industry, primarily of agricultural and food industry, for instance corn starch and raw glycerol (Ruhal and Choudhury 2012a). Some studies have indicated an improvement in the efficiency of obtaining trehalose by using osmotically sensitive mutants of *Propionibacterium* (Ruhal and Choudhury 2012a, b) and optimization of environmental conditions (Cardoso et al. 2004).

Cardoso et al. (2004) examined 18 strains of *Propionibacterium* isolated from different dairy sources for the production of trehalose. Among the studied microorganisms, only three did not exhibit the capacity to biosynthesize and accumulate trehalose. This indicates that the tested microorganisms have the capacity to produce trehalose. The highest level of accumulation of the trehalose disaccharide was determined in the cells of *P. freudenreichii* subsp*. shermanii* Nizo B365 (131 mg/g). Scientists (Cardoso et al. 2004) also determined the influence of the aforementioned strain on the environmental conditions during the production of trehalose. Lactose was found to be the best source of carbon for trehalose production. However, lactate, which has a very good impact on the growth of bacteria, was found to be a weak precursor of intracellular accumulation of the polysaccharide.

When the sources of carbon contained in the medium were depleted, Cardoso et al. (2004) started to observe a gradual loss of trehalose in the biomass, suggesting its role as a backup material in bacteria cells. They also determined an increase in intracellular accumulation of trehalose in the following conditions: environmental pH drop from 7.0 to 4.5, 2% NaCl concentration, and culture under aerobic conditions, where air saturation was 50%. The maximum trehalose accumulation in cells increased from 200 to 400 mg trehalose/g of biomass. These results indicate a dual role of trehalose in PAB. Being a reserve compound, this metabolite also functions to protect bacterial cell against environmental stress conditions. *P. freudenreichii* subsp*. shermanii* Nizo B365 can produce relatively large amounts of trehalose from reduced fat milk, which is promising with respect to the production of fermented dairy products enriched with trehalose (Cardoso et al. 2004).

Ruhal and Choudhury (2012a, b) demonstrated that trehalose biosynthesis by both mutant and wild-type *P. freudenreichii* subsp*. shermanii* in environment, where raw glycerol was the sole source of carbon. In case of the mutant with elevated osmotic sensitivity, accumulation of the metabolite was three times higher in comparison to the wild-type strain (it was 391 mg/g of biomass). This most likely stemmed from the increased activity of ADP-glucose pyrophosphorylase.

Industrial production of trehalose is primarily conducted by the enzymatic conversion (Chi et al. 2003). Although enzymatic methods are efficient, the use of bacteria and industrial waste in the production of trehalose has increased, which might influence the production cost of the trehalose disaccharide and therefore its market price (Li et al. 2011). The capability of certain bacteria to accumulate trehalose for the industrial application was tested for, among others, *Corynebacterium*, *E. coli* (Li et al. 2011), and *P. freudenreichii* (Ruhal et al. 2011; Ruhal and Choudhury 2012a, b; Dalmasso et al. 2012). Understanding the metabolism of trehalose might foster the development of strains resistant to stress, used in industrial fermentation processes. *Lactococcus lactis* is the best example in this sense. It is possible to develop a strain of the species characterized by increased trehalose accumulation and improved resistance to acid (pH 3.0), low temperature (4 °C), thermal shock (45 °C), and dehydration (Carvalho et al. 2011).

## Industrial application of trehalose

Trehalose was given GRAS status by the US FDA; therefore, it may be added to products intended for widely understood consumption (Mancini et al. 2011). Trehalose disaccharide has been widely used in the food, cosmetic, and pharmaceutical industry, and it has also been applied in medicine.

Trehalose is resistant to nonenzymatic browning, which is caused by the Maillard reaction (as a nonreducing sugar, trehalose is not subjected to reactions with compounds containing amino groups) and caramelization (this is due to the low energy of trehalose’s glycosidic bond, resulting in high durability). Trehalose is half as sweet as sucrose, ensures prolongation of energy levels, and causes very low insulin secretion. This makes trehalose disaccharide to be commonly used in the food production industries. It is primarily used as a sweetener and as a thickening (filling) agent; it masks unpleasant smells and protects starch, lipids, and proteins against oxidative damage and heat or cold damage. Trehalose is added to, for example, dried vegetables and fruits to maintain their aroma and organoleptic characters (Higashiyama 2002; Elbein et al. 2003; Kroger et al. 2006; Othake and Wang 2010). Considering the increasing consumer interest in terms of health and beauty, trehalose has a great potential in commercial applications, such as slimming sugar. An additional advantage of trehalose is in the fact that it exhibits a perfect stability during processing (Cardoso et al. 2004), as well as resistance to stress, such as low temperature and high osmolarity (Ruhal Choudhury 2012b). Due to the protective effect of trehalose on liposomes in cosmetics and proteins and lipids of the skin, it is in the preparation of moisturizing ointments. Research has demonstrated that 2% solution of trehalose can decrease unpleasant smells emitted by human skin up to 70% (it inhibits decomposition of unsaturated fatty acids and certain aldehydes). Therefore, trehalose finds application in deodorants, perfumes, and antiperspirants (Higashiyama 2002; Teramoto et al. 2008; Yu et al. 2012). It is a component of THEALOZ® drops used to treat dryness in the eye. This is because, it can protect corneal epithelial cells against dehydration and tissue denaturalization (Lee et al. 2013). Apart from this, trehalose is used in the production of solutions used in the cryopreservation of stem cells and storage of organs and tissues intended for transplantation. Moreover, it prolongs the shelf life of vaccines and antibodies, and it enables the storage of restrictive enzymes and DNA polymerase at room temperature. According to different studies, consuming meals containing trehalose influences the enhancement of bone metabolism and prevents development of osteoporosis (Higashiyama 2002; Teramoto et al. 2008; Yu et al. 2012). The so-called therapeutic proteins and polypeptides are used in the treatment of numerous diseases (e.g., arthritis, anemia, diabetes, and cancer). Their delicate structure makes them very susceptible to the effect of proteolytic enzymes, chemical and physical degradation, or aggregation in body fluids, all of these influence the loss of biological activity of the molecule. Research has been conducted (Jain and Roy 2008) on hydrogel protein carriers cross-linked with trehalose. Considering its bioprotective properties, when released, it will be capable of creating a microenvironment preventing inactivation of proteins and therapeutic polypeptides secreted alongside with trehalose. Moreover, attempts have been made to use trehalose in the treatment of neurodegenerative diseases such as Alzheimer’s, Parkinson’s, or Huntington’s disease. The atypical aggregation and fixation of individual proteins takes place in patients with any of the above diseases. A study demonstrated that trehalose decreases such effects and inhibits formation of neurotoxic amyloids (Liu et al. 2005; Miura et al. 2008; Yu et al. 2012; Chaudhary et al. 2014).

## Biosynthesis and role of bacteriocins synthesized by PAB

Bacteriocins are ribosome-synthesized peptide or protein molecules with an antimicrobial effect, which is produced by Gram-negative and Gram-positive bacteria. According to the literature, as much as 99% of the bacteria have the capability to biosynthesize at least one bacteriocin (Klaenhammer, 1993). It was initially thought that these substances exhibit activity solely toward microorganisms related to the producer of the given bacteriocin. However, subsequent research demonstrated that they have an effect on microorganisms of genera other than of the producer, including pathogenic microorganisms, such as *Listeria monocytogenes*, *Staphylococcus aureus*, and *Clostridium* spp. The activity, stability, and the way in which bacteriocins act are determined by their amino acid composition and molecular structure (Jack et al. 1995; Schillinger et al. 1996).

In recent years, consumers have expressed great interest in food without chemical preservatives; thus, a series of studies have been conducted on the biological methods of food preservation. One of the solutions might be bacterial metabolites exhibiting an antimicrobial effect, constituting an alternative for chemical preservatives, for instance bacteriocins or live bacteriocingenic cultures. These compounds are considered nontoxic to humans; they are sensitive to the effect of, among others, pepsin and trypsin (they are subjected to degradation in the digestive tract), and no negative impact of bacteriocins on organoleptic and sensory characters of food have been determined (Cabo et al. 2001; Cleveland et al. 2001). The application of bacteriocins as biopreservative in the food and feed industry requires understanding of characteristics of these compounds with particular emphasis on their structure, mechanism of action, and stability. Moreover, legalization of bacteriocins as food additives is necessary. Not without importance is also the economic issue related to the optimization of their production conditions. PAB are widely used in the production of food making them interesting as a potential source of compounds with antimicrobial properties (Faye et al. 2000; Miescher et al. 2000; Brede et al. 2004; Faye et al. 2004; van der Merwe et al. 2004; Gwiazdowska and Trojanowska 2005; Gwiazdowska 2010).

The classic and dermal bacteria of the genus *Propionibacterium* have the capability to produce bacteriocins. The majority of these compounds are produced by the classic species, such as *P. thoenii, P. jensenii*, and *P. freudenreichii* (Al-Zoreky et al. 1991; Faye et al. 2000; Miescher et al. 2000; Ben-Shushan et al. 2003; Brede et al. 2004; van der Merwe et al. 2004; Gwiazdowska and Trojanowska 2005).

Biosynthesis of bacteriocins depends on a variety of factors: pH, temperature, and composition and consistency of the culture medium. Appropriate pH for the production of bacteriocins by PAB depends on the strain being used. The production of most intensive propionicin PLG-1 takes place at a pH of 7, whereas the maximum production of jensenin G has been observed at pH 6.4 (Lyon et al. 1993; Hsieh et al. 1996; Ekinci and Barefoot 1999; Brede et al. 2004). Faye et al. (2000) observed a different optimum temperature during the biosynthesis of propionicin T1 using two strains of *P. thoenii*. The strain LMG 2792 attained highest production of propionicin T1at a temperature of 22 °C, whereas strain 419 attained its highest production at a temperature of 30 °C. The best media for the biosynthesis of bacteriocins by PAB under laboratory conditions are broth with sodium lactate, MRS, and medium consisting of beet molasses and corn steep. Ben-Shushan et al. (2003) demonstrated that the production of propionicin PLG-1 in a liquid medium by the *P. thoenii* P127 strain was higher than that of the GBZ-1 strain, whereas in semi-fluid medium, both bacteriocins had comparable production levels. Bacteriocins from *Propionibacterium* are characterized with low antimicrobial activity in culture environment. Therefore, it is important to optimize conditions related to the biosynthesis of bacteriocins and to optimize a method suitable for their separation and efficiency. Due to the low concentration of bacteriocins, it is necessary to concentrate postculture liquids to determine its antimicrobial activity (Lyon et al. 1993; Ekinci and Barefoot 1999; Morgan et al. 1999; Faye et al. 2000; Brede et al. 2004).

The mechanism of action of bacteriocins can be either bactericidal and/or bacteriostatic in nature. The majority of bacteriocins produced by PABs show an antagonistic effect toward *Propionibacterium* and *Lactobacillus* species. The effect of bacteriocins from PAB toward closely related strains is variable and depends on the degree of relationship of the producer’s strain and the sensitive strain. The activity of bacteriocins is further influenced by dose, degree of purification, growth phase of sensitive cells, pH, and temperature of the environment (Cintas et al. 2001). The majority of bacteriocins have bactericidal effect on sensitive microorganisms several minutes after contact. Certain bacteriocins (lactocin, leukocin) (Upreti 1994) have bacteriostatic effect, thus inhibiting the propagation of sensitive microorganisms. Bacteriocins may exhibit variable activity, depending on the sensitive microorganism; jensenina G has bactericidal effect against *L. delbrüecki* and bacteriostatic effect against *P. acidipropionici* (Grinstead and Barefoot 1992)*.* Contact of bacteriocin with sensitive cells takes place through electrostatic interactions between the positively charged molecule of inhibitor and negatively charged phospholipids (teichoic acid and lipoteichoic acid) found in the cellular membrane of the sensitive microorganism. Consequent to these interactions pores and ionic canals are formed in the cell membrane (certain bacteriocins require specific membrane receptors). The pores formed cause passive outflow of phosphate, potassium ions, amino acids, ATP, and other compounds, leading to the disturbance in the proton motor force, pH gradient, or membrane potential. Deficiency of ions, low ATP, and level of cofactor results in the inhibition of protein and nucleic acid synthesis; it is also impossible for cells to obtain nutrients from the environment, leading to their death (Bhunia et al. 1991; Jack et al. 1995; Chen et al. 1997; Marciset et al. 1997; Moll et al. 1999; Ryan et al. 1999). Certain bacteriocins disturb synthesis of cell wall via inhibition of biosynthesis of petidoglycans at the level of transglycosylation or induce the lysis of cells of sensitive bacterial species (deVuyst and Vandamme 1994; González et al. 1994).

The widest range of activity has been demonstrated for propionicin PLG-1 synthesized by *P. thoenii* P127 (Table 2), which has an antimicrobial effect toward PAB, lactic acid bacteria, and against Gram-negative bacteria, yeasts, and molds. Propionicin GBZ-1 and thoenicin 447 have an antibacterial effect toward the pathogenic strain of *P. acnes*. This suggests the possibility of using this compound in the treatment of acne caused by *P. acnes.* Jensenin G inhibits the growth of *Clostridium botulinum* spore types A, B, and E. Bacteriocins of selected PAB, such as propionicin F, propionicin T1, and propionicin SM1 have antagonistic effect only for strains of the same species as the producer. Nonpurified bacteriocin preparations containing propionicin SM1 exhibit antimicrobial activity against yeasts, molds, PAB, and lactic acid bacteria. After purification, propionicin SM1 exhibits activity solely toward the *P. jensenii* DSM20274 strain. This probably stems from the fact that a nonpurified preparation contains compounds supporting activity of the bacteriocin, which have antibacterial and fungistatic effect. In many cases, bacteriocins of PAB have stronger effect on lactic acid bacteria than on other PAB. Propionicin GBZ-1 has the strongest antagonist activity toward *Lactobacillus delbrueckii* subsp. *lactis* ATCC 4797. Thoenicin 447 has bactericidal effect toward *Lactobacillus delbrueckii* subsp. *bulgaricus* LMG 13551, whereas it shows bacteriostatic effect toward *P. acnes*. Jensenin G is characterized by elevated activity toward genera *Lactobacillus* and *Lactococcus*. Enzymatically activated bacteria are characterized by antagonism toward strains of *P. acidipropionici*, *P. freudenreichii*, *P. jensenii*, *P. thoenii*, and six strains of the genus *Lactobacillus* (Lyon and Glatz 1991; Ratnam et al. 1999; Faye et al. 2000; Miescher et al. 2000; Faye et al. 2002; Ben-Shushan et al. 2003; Brede et al. 2004; van der Merwe et al. 2004).

**Table 2:** Characteristics of selected bacteriocins of propionic acid bacteria

Bacteriocins	Molecular weight (Da)	Stability		Inactivating enzymes	Range of activity	References
		pH	Temp.			
Propionicin PLG-1	9238	3–9	80 °C below 15 min	Protease, pronase E, pepsin, trypsin, α-chymotrypsin	Bacteria G*(+): Lactobacillus bulgaricus*, *L. casei*, *Pediococcus cervisiae* i inne, bakterie G(-): *Campylobacter jejuni*, *Escherichia coli*, *Vibrio parahaemolyticus*, and other, fungi: *Aspergillus wentii* (ATCC 1778), *Apiotrichum curvatum*, *Candida utilis*, *C. lipolytica* and other	Lyon and Glatz 1991; van der Merwe et al. 2004
Propionicin T1	7130.20	> 2.5	60–100 °C below 15 min	Proteinase K	*P. acidipropionici* ATCC 4965, ATCC 4875, *P. jensenii* ATCC 4868, P17, P52, *P. thoenii* ATCC 4871, ATCC 4872 *Lactobacillus sakei* NCDO 2714 and other	Faye et al. 2000
Propionicin F	4397	No data	No data	Proteinase K	strains of the species *Propionibacterium freudenreichii*	Brede et al. 2004
Thoenicin 447	7130	1–10	100 °C below 15 min	Proteinase K, pronase, pepsin, trypsin, α -chymotrypsin	*Lactobacillus bulgaricus* LMG 13551, *Propionibacterium acnes* ATCC 6919, ATCC 6922, ATCC 11827, ATCC 11828	van der Merwe et al. 2004
Propionicin SM1	19.942	No data	No data	No data	*P. jensenii* DSM 20274, DSM 20535	Miescher et al. 2000
Jensenin G	> 12.000	No data	100 °C by 2 min	Proteinase K, pronase E, protease	*P. acidipropionici* P5, *P. jensenii* P54, *Lactobacillus bulgaricus* NCDO 1489, *L. delbrueckii subsp. lactis* ATCC 4797, *Clostridium botulinum* type A, B, E and other	Sip et al. 2009
Jensenin P	6000–9000	3–12	100 °C by 60 min	No data	*P. jensenii* B1264*, P. thoenii* P127 *P. acidipropionici* P5, *P. thoenii* P126, *Lactobacillus acidophilus* ATCC 4356, *L. delbruecki* subsp. *delbruecki* ATCC9649 and other	Ratnam et al. 1999
PAMP	6383	No data	No data	In concentration > 100 μg/ml: proteinase K, A, P, trypsin,	selected strains of species: *P. acidipropionici, P. freudenreichii*, *P. jensenii*, *P. thoenii*, bacteria of the genus *Lactobacillus*	Faye et al. 2000

## Characteristics of selected bacteriocins

The smallest currently known bacteriocin synthesized by *Propionibacterium* is propionicin F. Its molecular mass is 4397 Da, and its mature form consists of 47 amino acids. It is the first bacteriocin that has been isolated from the culture of two different strains of the species *P. freudenreichii*: LMGT 2956 and LMGT 2946 (Brede et al. 2004). It is coded by the *pcfA* gene, below which genes cotranscribed with *pcfA* are found: *pcfB*, *pcfC*, and *pcfD*. It is suspected that propionicin F is activated by two processes: the first process includes the cutoff of 101 amino acids from the N-terminal part and the second consists of the removal of 111 amino acids from the C-terminal part of the propeptide. Propropionicin F contains cisteins located in the cutoff site of the amino end of the probacteriocin; therefore, according to Brede et al. (2004), the *pcfB* gene encoding S-adenosylmethionine transferase participates in the release of the N-terminal part with the concomitant formation of cysteine residue. The cutoff of the C-terminal part is attributed to the *pcfC* gene, which codes proline aminopeptidase (Brede et al. 2005; Faye et al. 2011). The presence of proline in the carboxylic region of proline peptidase might participate in the posttranslational processing of the bacteriocin. It is an outsized process, as it consists of the cutoff of fragments on both ends of probacteriocin, and literature provides with cases of posttranslational processing of primarily one end. The genes *pcfD* (coding ABC transporter) and *pcfC* are responsible in the secretory system of the bacteriocin outside the cell. Membrane proteins coded by *pcfI* control the resistance of cells against propionicin F (Brede et al. 2007).

Propionicin SM1 is the largest of the currently known bacteriocins of PAB produced by two different strains of *P. jensenii*. It is formed as a 207-amino acid propeptide. As for the majority of bacteriocins, propionicin SM1 is secreted outside of a cell when a 27-amino acid signal fragment of the N-terminal end is cut off. The signal peptide has typical structure and possesses positively charged amino end, central hydrophobic region, and polar carboxylic end (von Heijne 1988).

Propionicin T1 is a positively charged bacteriocin with a molecular mass of 7.1 kDa. It demonstrates activity against the following bacteria: *P. acidipropionici*, *P. jensenii*, *P. thoenii*, and *P. acnes* (US patent no. 2003/0096365A1) and certain lactic acid strains. Based on the identification of the propionicin T1 encoding gene (*pctA*), it has been determined that propionicin T1 is synthesized as a 96 amino acid prepeptide containing 31 amino acid long leader peptide on its amino end characterized by typical features of signal peptide, such as positively charged amino end, hydrophobicity, and specific cutoff region. As a result of translocation and processing by sec-system, prepeptide is transformed into active, 65 amino acid bacteriocin. DNA analysis revealed protein coding region (*orf2*) located at the distance of 68 nucleotides below the stop codon of the structural gene *pctA*; the amino end of this region indicates its bonding with ABC transporters. This protein is located in the plasma membrane and possesses four ATP-binding sites in the cytoplasm. As propionicin T1 exhibits characteristics of proteins secreted with the basic secretion method (von Heijne 1988), an ATP-binding cassette (ABC) transporter probably is not linked with its transport outside of a cell. The gene location suggests that the ABC transporter is responsible for the resistance of the cell to propionicin T1, that is, removal of active bacteriocin outside of a cell or its import and degradation inside the cell (Faye et al. 2000). ABC transporters protect producers against nisin, subtilisin, and lacticin 481 (Klein and Entian, 1994; Siegers and Entian, 1995; Rince et al. 1997). Bacteriocin with a fully homologous sequence to that of propionicin T1 is a thoenicin 447 produced by *P. thoenii* 447. Its mature form consists of 65 amino acids. The amino acid composition of thoenicin 447 contains a typical pattern of signal peptide (Nielsen et al. 1997).

A special type of protein produced by *Propionibacterium* is the so-called pathogen associated molecular patterns (PAMPs). The name refers to cell-produced proteins similar to bacteriocins, which are activated only as a result of protease activity. They were first described by Ratnam et al. (1999). The authors introduced an atypical manner of action of compounds produced by the following strains: *P. jensenii* B1264, *P. jensenii* 4868, and *P. freudenreichii* 6207. *P. jensenii* B1264, with the absence of proteolytic enzymes in the culture environment, exhibited activity solely toward propionic bacteria and *Lactobacillus delbrueckii*. Due to protease activity, the scope of PAMP activity of the strain was extended to *Lactococcus lacti*s subsp. *lactis* C2, *Lactobacillus helveticus* ATCC 15009, and *Lb. plantarum* PI 549. *P. jensenii* ATCC 4868 and *P. freudenreichii* 6207 with the absence of proteolytic enzymes, had an effect only on PAB, whereas in the presence of proteases they inhibited the growth of *Lactobacillus*. Faye et al. (2002) characterized PAMPs produced by *P. jensenii* LMG 3032 and *P. jensenii* ATCC 4868.

PAMP is produced as a propeptide consisting of 198 amino acids, with a 27-amino acid-long signal peptide located on the N-end. The mature form of PAMP contains a fragment of C-end of this precursor. PAMP activation takes place only outside the cell, which is rather atypical with reference to other bacteriocins. The majority of bacteriocins are produced in the form of peptide precursors and then subjected to intracellular posttranslational modifications or during protein translocation outside of the cell, and then they are transformed into biologically active forms. The pro-PAMP amino acid sequence indicates the presence of cutoff sites for example, proteinase K (specific region located between two arginines). PAMP exposed to the effect of proteinase K at a concentration of 40 μg/mL results in the formation of active bacteriocin with a molecular mass of 6383 Da (Neumann et al. 1993; Faye et al. 2002).

Another inhibiting substance (BLIS) produced by PAB, precisely by *P. jensenii* B1264 strain, has been identified (Wang et al. 2014). BLIS inhibits the growth of *P. acnes* ATCC 6919; thus, the protein might have a new potential application in the pharmaceutical industry to combat acne.

## Perspectives for the application of PAB bacteriocins

The majority of bacteriocins synthesized by classic PAB are classified as low-molecular weight proteins, the molecular mass of which does not exceed 10.000 Da, with the exception for propionicin SM1, whose mass is 20.000 Da. Bacteriocins are characterized by high stability in a wide range of pH and temperature ranges. They are active in acidic, neutral, and alkaline environment and are thermostable, not losing activity after several or 15-min-long heating at 100 °C. As an example, activity of thoenicin 447 after 15-min heating at 100 °C remains stable; the activity drops (by 80%) only at 121 °C. Jensenin G remains active for up to 2 min of heating at 100 °C, but longer exposure to such temperature (5, 10, and 15 min) limits its activity, but without degradation. Certain PAB bacteriocins, such as jensenin P, are stable in environment containing 0.1–1 mol/l NaCl, 0.1–2.0% SDS, 4 mol/l urea, and in the presence of different organic solvents. Propionicin PLG-1 and other bacteriocins are resistant to deterioration upon prolonged storage, retaining activity for up to 25 weeks (in temperatures of 4 and −25 °C). All these characteristics prove the usefulness of PAB bacteriocins in the food production and preservation (Hsieh et al. 1996; Ratnam et al. 1999; Ben-Shushan et al. 2003). The activity of the aforementioned bacteriocins, directed at lactic acid bacteria, can be used in the production of fermented dairy products to prevent excessive development of lactic acid bacteria, which causes excessive acidification of the product. Similar research has been conducted for, among others, jensenin G (Weinbrenner et al. 1997). Bacteriocins have also been tested for meat preservation. Ekİncİ and Candoğan (2014) evaluated the influence of a mixture of jensenin G and ethylenediaminetraacetic acid (EDTA) toward *Listeria monocytogenes* and *E. coli* in pieces of fresh meat. A combination of jensenin G and EDTA resulted in the decrease of the number of *Listeria* bacilli found in beef from 4.78 to 3.49 log cfu/g, but it did not have a significant effect on the reduction of *E. coli.* Divek and Kollanoor-Johny (2016) tested the influence of two subspecies of *P. freudenreichii* (*P. freudenreichii* subsp. *freudenreichii* and *P. freudenreichii* subsp*. shermanii*) toward three bacterial species of the genus *Salmonella* (*S. enteritidis*, *S. typhimurium*, and *S. Heidelberg)*. Both *P. freudenreichii* strains proved to be efficient in inhibiting the propagation of all three species. Apart from this, *P. freudenreichii* had significant influence on the decrease of mobility of *Salmonella*. PBA bacteriocins could also be applied in cheese making, in the restriction of formation of red spots on cheeses caused by the pigment produced by *P. thoenii* and *P. jensenii* without any negative effect on starter cultures of *P. freudenreichii* (Sip et al. 2009)*.* The antibacterial activity of certain bacteriocins toward *P. acnes* creates the potential for the application of such proteins as additives to anti-acne preparations (Wang et al. 2014). It appears that in the future, bacteriocins produced by *Propionibacterium* might be widely used as inhibiting agents against the development of undesirable microorganisms in a variety of food, cosmetic, and pharmaceutical products.

## Conclusion

PAB, owing to their considerable potential, are attracting increasing attention of researchers and different branches of feed, food, pharmaceutical, and medical industries. These bacteria possess a complex enzymatic system, enabling them to utilize a wide array of carbon sources. However, the most important fact—in the context of industrial use of propionic acid bacteria—is that they are capable of producing numerous biologically active compounds, including propionic acid, vitamin B12, trehalose, and bacteriocins from by-products of technological processes. Thus, the biotechnological use of PAB might help to decrease the environmental pollution by transforming the waste into usable and valuable components for other industries. However, further research is necessary to increase the biosynthetic efficiency of these metabolites so that the production can be scaled up, for instance with the use of waste products, which might be more cost-effective than the current production methods. Moreover, further studies on the biosynthesis of metabolites by the bacteria from the *Propionibacterium* genus are needed, associated with the optimization of culture conditions for example, by addition to the culture medium suitable carbon sources, biostimulators, or genetic modifications. However, because in many parts of the world, genetically modified organisms (GMOs) still cause many problems, the search for appropriate microbes capable of producing this metabolite with high efficiency without interfering with their genome has intensified.

## References

[CR1] Aleman G, Tovar AR, Torres N (2001). Homocysteine metabolism and risk of cardiovascular diseases: importance of the nutritional status on folic acid, vitamins B6 and B12. Rev Investig Clin.

[CR2] Al-Zoreky NJ, Ayres W, Sandine WE (1991). Antimicrobial activity of Microgard™ against food spoilage and pathogenic microorganisms. J Dairy Sci.

[CR3] Ammar EM, Jin Y, Wang Z, Yang ST (2014). Metabolic engineering of *Propionibacterium freudenreichii*: effect of expressing phosphoenolpyruvate carboxylase on propionic acid production. Appl Microbiol Biotechnol.

[CR4] Angelopoulou A, Alexandraki V, Georgalaki M, Anastasiou R, Manolopoulou E, Tsakalidou E, Papadimitriou K (2017). Production of probiotic Feta cheese using *Propionibacterium freudenreichii* subsp. *shermanii* as adjunct. Int Dairy J.

[CR5] Avonce N, Mendoza-Vargas A, Morett E, Iturriaga G (2006). Insights on the evolution of trehalose biosynthesis. BMC Evol Biol.

[CR6] Barbirato F, Chedaille D, Bories A (1997). Propionic acid fermentation from glycerol: comparison with conventional substrates. Appl Microbiol Biotechnol.

[CR7] Baumann I, Westermann P (2016) Microbial production of short chain fatty acids from lignocellulosic biomass: current processes and market, Hindawi publishing Corporation. BioMed Res Int 2016:1–15. 10.1155/2016/8469357 10.1155/2016/8469357 PMC4983341 27556042

[CR8] Beck WS, Rucker RB, Suttie JW, McCormick DB, Machlin LJ (2001). Cobalamin (Vitamin B12). Handbook of Vitamins.

[CR9] Ben-Shushan G, Zakin V, Gollop N (2003). Two different propionicins produced by *Propionibacterium thoenii* P-127. Peptides.

[CR10] Benaroudj N, Lee DH, Goldberg AL (2001). Trehalose accumulation during cellular stress protect cells and cellular proteins from damage by oxygen radicals. J Biol Chem.

[CR11] Benjelloun H, Rabe Ravelona M, Lebeault JM (2007). Characterization of growth and metabolism of commercial strains of propionic acid bacteria by pressure measurement. Eng Life Sci.

[CR12] Bhunia AK, Johnson MC, Ray B, Kalchayanad N (1991). Mode of action of pediocin AcH from *Pediococcus acidilactici* H on sensitive bacterial strains. J Appl Bacteriol.

[CR13] Blanche F, Debussche L, Thibaut D, Crouzet J, Cameron B (1989). Purification and characteriztationof S-adenosyl-L-methionine: uroporphyrinogen III methyltransferase from *Pseudomonas denitrificans*. J Bacteriol.

[CR14] Blanche F, Cameron B, Crouzet J, Debussche L, Thibaut D, Vuilhorgne M, Lepper FJ, Battersby AR (1995). Vitamin B12: how the problem of its biosynthesis was solved. Angew Chem Int Ed Engl.

[CR15] Blanche F, Cameron B, Crouzet J, Debussche L, Levy-Schil S, Thibaut D (1998). Rhône- Poulenc Biochimie. Eur Patent.

[CR16] Borawska J, Warmińska-Radyko I, Darewicz M (2010). Charakterystyka i znaczenie bakterii rodzaju *Propionibacterium* w produkcji żywności i pasz. Med Wet.

[CR17] Boyaval P, Corre C (1987). Continuous fermentation of sweet whey permeate for propionic acid production in a CSTR with UF recycle. Biotechnol Lett.

[CR18] Boyaval P, Deborde C, Corre C, Blanco C, Bégué É (1999). Stress and osmoprotection in propionibacteria. Lait.

[CR19] Brede DA, Faye T, Johnsborg O, Ødegård I, Nes IF, Holo H (2004). Molecular and genetic characterization of propionicin F, a bacteriocin from *Propionibacterium freudenreichii*. Appl Environ Microbiol.

[CR20] Brede DA, Faye T, Stierli MP, Dasen G, Theiler A, Nes IF, Meile L, Holo H (2005). Heterologous production of antimicrobial peptides in *Propionibacterium freudenreichii*. Appl Environ Microbiol.

[CR21] Brede DA, Lothe S, Salehian Z, Faye T, Nes IF (2007). Identification of the propionicin F bacteriocin immunity gene (*pcfI*) and development of a food-grade cloning system for *Propionibacterium freudenreichii*. Appl Environ Microbiol.

[CR22] Breed RS, Murray EGD, Smithh NR (1957) Bergey’s manual of determinative bacteriology. Seventh edition. The Williams & Wilkins Company, pp 1346-1353

[CR23] Cabo ML, Murado MA, González MP, Pastoriza L (2001). Effects of aeration and pH gradient on nisin production. A mathematical model. Enzym Microb Technol.

[CR24] Cánovas D, Fletcher SA, Hayashi M, Csonka LN (2001). Role of trehalose in growth at high temperature of *Salmonella enterica* serovar *typhimurium*. J Bacteriol.

[CR25] Cardoso S, Gaspar P, Hugenholtz J, Ramos A, Santos H (2004). Enhancement of trehalose production in dairy propionibacteria through manipulation of environmental conditions. Int J Food Microbiol.

[CR26] Cardoso FS, Castro RF, Borges N, Santos H (2007). Biochemical and genetic characterization of the pathways for trehalose metabolism in *Propionibacterium freudenreichii*, and their role in stress response. Microbiology.

[CR27] Carrondo MJ, Crespo JP, Moura M (1988). Production of propionic acid using a xylose utilizing *Propionibacterium*. Appl Biochem Biotechnol.

[CR28] Carvalho AL, Cardoso FS, Bohn A, Neves AR, Santos H (2011). Engineering trehalose synthesis in *Lactococcus lactis* for improved stress tolerance. Appl Environ Microbiol.

[CR29] Chamlagain B, Deptula P, Edelmann M, Kariluoto S, Grattepanche F, Lacroix C, Varmanen P, Piironen V (2016). Effect of the lower ligand precursors on vitamin B12 production by food-grade Propionibacteria. LWT - Food Sci Technol.

[CR30] Chamlagain B (2016). Fermentation fortification of active vitamin B12 in food matrices using Propionibacterium freudenreichii: analysis, production and stability.

[CR31] Chaudhary RK, Kardani J, Singh K (2014). Deciphering the roles of trehalose and Hsp104 in the inhibition of aggregation of mutant huntingtin in a yeast model of Huntington’s disease. NeuroMolecular Med.

[CR32] Chen Y, Shapira R, Einstein M, Montville TJ (1997). Functional characterization of pediocin PA-1 binding to liposomes in the absence of a protein receptor and its relationship to a predicted tertiary structure. Appl Environ Microbiol.

[CR33] Chen F, Feng XH, Xu H, Zhang D, Ouyang PK (2012). Propionic acid production in a plant fibrous-bed bioreactor with immobilized *Propionibacterium freudenreichii* CCTCC M207015. J Biotechnol.

[CR34] Chi Z, Liu J, Zhang W (2001). Trehalose accumulation from soluble starch by *Saccharomycopsis fibuligera* sdu. Enzyme Microb Tech.

[CR35] Chi Z, Liu J, Ji J, Meng Z (2003). Enhanced conversion of soluble starch to trehalose by a mutant of *Saccharomycopsis fibuligera* sdu. J Biotech.

[CR36] Chiliveri SR, Yeruva T, Panda SH, Linga VR (2010) Optimization of fermentation parameters for vitamin B12 production using *Propionibacterium freudertreichii* subsp. *shermanii* OLP-5 by Taguchi method. J Pure Appl Microbiol 4:647–658

[CR37] Cintas LM, Casaus P, Herranz C, Nes IF, Hernandez PE (2001). Review: bacteriocins of lactic acid bacteria. Food Sci Tech Int.

[CR38] Cleveland J, Montville T, Nes IF, Chikindas M (2001). Bacteriocins: safe, natural antimicrobials for food preservation. Int J Food Microbiol.

[CR39] Cong L, Ran FA, Cox D, Lin S, Barretto R, Habib N, Hsu PD, Wu X, Jiang W, Marraffini LA (2013). Multiplex genome engineering using CRISPR/Cas systems. Science.

[CR40] Cousin FJ, Jouan-Lanhouet S, Théret N (2016). The probiotic *Propionibacterium freudenreichii* as a new adjuvant for TRAIL-based therapy in colorectal cancer. Oncotarget.

[CR41] Crofts TS, Seth EC, Hazra AB, Taga ME (2013). Cobamide structure depends on both lower ligand availability and CobT substrate specificity. Chem Biol.

[CR42] Cybulska J, Zdunek A, Sitkiewicz I, Galus S, Janiszewska E, Łaba S, Nowacka M (2013). Możliwości zagospodarowywania wytłoków i innych odpadów przemysłu owocowo-warzywnego. Przem Ferm i Owoc-Warzyw.

[CR43] Czaczyk K, Trojanowska K, Stachowiak B (1997). The effect of immobilization of propionic acid bacteria in alginate gel on the course of fermentation. Pol J Food Nutr Sci.

[CR44] Czaczyk K, Trojanowska K, Grajek W (1997). The influence of a specific microelemental environment in alginate gel beads on the course of propionic acid fermentation. Appl Microbiol Biotechnol.

[CR45] Dalmasso M, Aubert J, Briard-Bion V, Chuat V, Deutsch SM, Even S, Falentin H, Jan G, Jardin J, Maillard MB, Parayre S, Piot M, Tanskanen J, Thierry A, Gilbert A (2012) A Temporal -omic Study of Propionibacterium freudenreichii CIRM-BIA1T Adaptation Strategies in Conditions Mimicking Cheese Ripening in the Cold. PLoS ONE 7(4):1–15. 10.1371/annotation/e0ff065d-a52d-44f2-8727-328393ed60b6 10.1371/journal.pone.0029083 PMC3258244 22253706

[CR46] Daly DFM, McSweeney PLH, Sheehan JJ (2010). Split defect and secondary fermentation in Swiss-type cheeses: a review. Dairy Sci Technol.

[CR47] Deptula P, Kylli P, Chamlagain B, Holm L, Kostiainen R, Piironen V, Savijoki K, Varmanen P (2015). BluB/CobT2 fusion enzyme activity reveals mechanisms responsible for production of active form of vitamin B_12_ by *Propionibacterium freudenreichii*. Microb Cell Factories.

[CR48] De Smet KAL, Weston W, Brown IN, Young D, Robertson BD (2000). Three pathways for trehalose biosynthesis in mycobacteria. Microbiology.

[CR49] de Virgilio C, Hottiger T, Dominguez J, Boller T, Wiemken A (1994). The role of trehalose synthesis for the acquisition of thermotolerance in yeast. FEBS J.

[CR50] De Vuyst L, Vandamme EJ, de Vuyst L, Vandamme EJ (1994). Antimicrobial potential of lactic acid bacteria. Bacteriocins of lactic acid bacteria.

[CR51] Dharmarajan TS, Adiga GU, Norkus EP (2003). Vitamin B12 deficiency. Recognizing subtle symptoms in older adults. Geriat.

[CR52] Divek VTN, Kollanoor-Johny A (2016). Effect of *Propionibacterium freudenreichii* on salmonella multiplication, motility, and association with avian epithelial cells1. Poult Sci.

[CR53] Ekİncİ FY, Barefoot SF (1999). Production of bacteriocin jenseniin G by *Propionibacterium* is pH sensitive. Lett Appl Microbiol.

[CR54] Ekİncİ FY, Candoğan K (2014). Control of foodborne pathogens on fresh beef by jenseniin G, a bacteriocin produced by *Propionibacterium thoenii* (*jensenii*) P126. Ar Latinoam Prod Animal.

[CR55] Elbein AD, Pan YT, Pastuszak I, Carroll D (2003). New insights on trehalose: a multifunctional molecule. Glycobiology.

[CR56] Erickson LE, Minkevich IG, Eroshin VK (1979). Utilization of mass-energy balance regularities in the analysis of continuous-culture data. Biotechnol Bioeng.

[CR57] Falentin H, Deutsch SM, Jan G, Loux V, Thierry A, Parayre S, Maillard MB, Dherbécourt J, Cousin FJ, Jardin J, Siguier P, Couloux A, Barbe V, Vacherie B, Wincker P, Gibrat JF, Gaillardin C, Lortal S (2010). The complete genome of *Propionibacterium freudenreichii* CIRM-BIA1^T^, a hardy Actinobacterium with food and probiotic applications. PLoS One.

[CR58] Faye T, Langsrud T, Nes IF, Holo H (2000). Biochemical and genetic characterization of propionicin T1, a new bacteriocin from *Propionibacterium thoenii*. Appl Environ Microbiol.

[CR59] Faye T, Brede DA, Lansgrud T, Nes IF, Holo H (2002). An antimicrobial peptide is produced by extracellular processing of a protein from *Propionibacterium jensenii*. J Bacteriol.

[CR60] Faye T, Lansgrud T, Nes IF, Holo H (2004). Prevalence of the genes encoding Propionicin T1 and protease-activated antimicrobial peptide and their expression in classical Propionibacteria. Appl Environ Microbiol.

[CR61] Faye T, Holo H, Langsrud T (2011). The unconventional antimicrobial peptides of the classical propionibacteria. Appl Microbiol Biotechnol.

[CR62] Feng X, Chen F, Xu H, Wu B, Li H, Li S, Ouyang P (2011). Green and economical production of propionic acid by *Propionibacterium freudenreichii* CCTCC M207015 in plant fibrous-bed bioreactor. Bioresour Technol.

[CR63] Fenech M (2001). The role of folic acid and vitamin B12 in genomic stability of human cells. Mutat Res.

[CR64] Figlin E, Chetrit A, Shahar A, Shpilberg O, Zivelin A, Rosenberg N, Brok-Simoni F, Gadoth N, Sela BA, Seligsohn U (2003). High prevalences of vitamin B12 and folic acid deficiency in elderly subjects in Israel. Br J Haematol.

[CR65] Fröhlich-Wyder MT, Bachmann HP, Casey MG (2002). Interaction between propionibacteria and starter/non-starter lactic acid bacteria in Swiss-type cheeses. Lait.

[CR66] González B, Arca P, Mayo B, Suárez JE (1994). Detection, purification, and partial characterization of plantaricin C, a bacteriocin produced by a *Lactobacillus plantarum* strain of dairy origin. Appl Environ Microbiol.

[CR67] Gardner N, Champagne CP (2005). Production of *Propionibacterium shermanii* biomass and vitamin B12 on spent media. J Appl Microbiol.

[CR68] Grinstead DA, Barefoot SF (1992). Jenseniin G, a heat-stable bacteriocin produced by *Propionibacterium jensenii* P126. Appl Environ Microbiol.

[CR69] Guan N, Liu L, Zhuge X, Xu Q, Li J, Du C, Chen J (2012). Genome shuffling improves acid tolerance of *Propionibacterium acidipropionici* and propionic acid production. Adv Chem Res.

[CR70] Guan N, Liu L, H-d S, Chen RR, Zhang J, Li J, Du G, Shi Z, Chen J (2013). Systems-level understanding how *Propionibacterium acidipropionici* respond to propionic acid stress at the microenvironment levels: mechanism and application. J Biotechnol.

[CR71] Guan N, Shin HD, Chen RR, Li J, Liu L, Du G, Chen J (2014). Understanding of how *Propionibacterium acidipropionici* respond to propionic acid stress at the level of proteomics. Sci Rep.

[CR72] Guan N, Li J, Shin H, Du G, Chen J, Liu L (2015). Metabolic engineering of acid resistance elements to improve acid resistance and propionic acid production of *Propionibacterium jensenii*. Biotechnol Bioeng.

[CR73] Guan N, Li J, Shin H, Wu J, Du G, Shi Z, Liu L, Chen J (2015). Comparative metabolomics analysis of the key metabolic nodes in propionic acid synthesis in *Propionibacterium acidipropionici*. Metabolomics.

[CR74] Guan N, Zhuge X, Li J (2015). Engineering propionibacteria as versatile cell factories for the production of industrially important chemicals: advances, challenges, and prospects. Appl Microbiol Biotechnol.

[CR75] Gwiazdowska D, Trojanowska K (2005). Bakteriocyny – właściwości i aktywność przeciwdrobnoustrojowa. Biotechnologia.

[CR76] Gwiazdowska D (2010). Biochemiczna i molekularna charakterystyka bakteriocyn wytwarzanych przez bakterie z rodzaju *Propionibacterium*. Biotechnologia.

[CR77] Hatanaka H, Wang E, Taniguchi M, Iijima S, Kobayashi T (1998). Production of vitamin B12 by a fermentor with a hollow-fiber module. Appl Microbiol Biotechnol.

[CR78] Higashiyama T (2002). Novel functions and applications of trehalose. Pure Appl Chem.

[CR79] Himmi E, Bories A, Boussaid A, Hassani L (2000). Propionic acid fermentation of glycerol and glucose by Propionibacterium acidipropionici and *Propionibacterium freudenreichii* ssp. *shermanii*. Appl Microbiol Biotechnol.

[CR80] Hojo K, Watanabe R, Mori T, Taketomo N (2007). Quantitative measurement of tetrahydromenaquinone-9 in cheese fermented by propionibacteria. J Dairy Sci.

[CR81] Hsieh H, Paik H, Glatz B (1996). Improvement of detection and production of propionicin PLG-1, a bacteriocin produced by *Propionibacterium thoenii*. J Food Protect.

[CR82] Hsu ST, Yang ST (1991). Propionic acid fermentation of lactose by *Propionibacterium acidipropionici*: effects of pH. Biotechnol Bioeng.

[CR83] Huang YL, Wu Z, Zhang L, Ming Cheung C, Yang ST (2002). Production of carboxylic acids from hydrolyzed corn meal by immobilized cell fermentation in a fibrous-bed bioreactor. Bioresour Technol.

[CR84] Hugenholtz J, Hunik J, Santos H, Smid E (2002). Nutraceutical production by propionibacteria. Lait.

[CR85] Jack RW, Tagg JR, Ray B (1995). Bacteriocins of gram-positive bacteria. Microbiol Rev.

[CR86] Jan G, Leverrier P, Proudy I, Roland N (2002). Survival and beneficial effects of propionibacteria in the human gut: in vivo and in vitro investigations. Lait.

[CR87] Jain NK, Roy I (2008). Effect of trehalose on protein structure. Protein Sci.

[CR88] Jiang W, Bikard D, Cox D, Zhang F, Marraffini LA (2013). RNA-guided editing of bacterial genomes using CRISPR-Cas systems. Nat Biotechnol.

[CR89] Jin Z, Yang ST (1998). Extractive fermentation for enhanced propionic acid production from lactose by *Propionibacterium acidipropionici*. Biotechnol Prog.

[CR90] Jinek M, Chylinski K, Fonfara I, Hauer M, Doudna JA, Charpentier E (2012). A programmable dual-RNA-guided DNA endonuclease in adaptive bacterial immunity. Science.

[CR91] Jordan PM (1994). Highlights in haem biosynthesis. Curr Opin Struct Biol.

[CR92] Khan Mazharuddin M, Mir NA, Khan M (2011). Production of vitamin B_12_ by improved strains of *Propionibacterium freudenreichii*. Biotechnol Bioinf Bioeng.

[CR93] Klaenhammer TR (1993). Genetics of bacteriocins produced by lactic acid bacteria. FEMS Microbiol Rev.

[CR94] Klein C, Entian KD (1994). Genes involved in self-protection against the lantibiotic subtilin produced by *Bacillus subtilis* ATCC 6633. Appl Environ Microbiol.

[CR95] Knasmüller S, Verhagen H (2002). Impact of dietary on cancer causes and DNA integrity: new trends and aspects. Food Chem Toxicol.

[CR96] Kolling K, Ndrepepa G, Koch W, Braun S, Mehilli J, Schoming A, Kastrati A (2004). Methylenetetrahydrofolate reductase gene C677T and A1298C polymorphism, plasma homocysteine, folate and vitamin B12 levels and the extent of coronary artery disease. Am J Cardiol.

[CR97] Kośmider A, Drożdżyńska A, Blaszka K, Leja K, Czaczyk K (2010). Propionic acid production by *Propionibacterium freudenreichii* ssp. *shermanii* using crude glycerol and whey lactose industrial wastes. Polish J of Environ Stud.

[CR98] Kośmider A, Czaczyk K (2010). Witamina B_12_ – budowa, biosynteza, funkcje i metody oznaczania. ŻNTJ.

[CR99] Kourkoutas Y, Xolias V, Kallis M, Bezirtzoglou E, Kanellaki M (2005). *Lactobacillus casei* cell immobilization on fruit pieces for probiotic additive, fermented milk and lactic acid production. Process Biochem.

[CR100] Koussémon M, Combet-Blanc Y, Ollivier B (2003). Glucose fermentation by *Propionibacterium microaerophilum*: effect of pH on metabolism and bioenergetics. Curr Microbiol.

[CR101] Kroger M, Meister K, Kava R (2006). Low-calorie sweeteners and other sugar substitutes: a review of the safety issues. Compr Rev Food Sci F.

[CR102] Kujawski M, Rymaszewski J, Cichosz G, Łaniewska-Moroz Ł, Fetliński A (1994). Wpływ bakterii fermentacji propionowej na tworzenie cech smakowo-zapachowych sera i twarogów. Przegl Mlecz.

[CR103] Kusano K, Yamada H, Niwa M, Yamasato K (1997). *Propionibacterium cyclohexanicum* sp. nov., a new acid-tolerant ω-Cyclohexyl fatty acid-containing *Propionibacterium* isolated from spoiled orange juice. Int J Sys Evol Microb.

[CR104] Lamm L, Heckman G, Renz P (1982). Biosynthesis of vitamin BI2 in anaerobic bacteria, mode of incorporation of glycine into the 5,6-dimethylbenzimidazole moiety in Eubacteviumlimosum. Eur J Biochem.

[CR105] Langsrud T, Sorhauq T, Vegarud GE (1995). Protein degradation and amine acid metabolism by propionibacteria. Lait.

[CR106] Lee J, Lin EW, Lau UY, Hedrick JL, Bat E, Maynard HD (2013). Trehalose glycopolymers as excipients for protein stabilization. Biomacromolecules.

[CR107] Leman J, Bednarski W, Fiedurek J (2007). Podstawy technologii wybranych bioproduktów. Podstawy biotechnologii przemysłowej.

[CR108] Leverrier P, Vissers JPC, Rouault A, Boyaval P, Jan G (2004). Mass spectrometry proteomic analysis of stress adaptation reveals both common and distinct response pathways in *Propionibacterium freudenreichii*. Arch Microbiol.

[CR109] Lewis VP, Yang ST (1992). A novel extractive fermentation process for propionic acid production from whey lactose. Biotechnol Prog.

[CR110] Li HW, Zang BS, Deng XW (2011). Overexpression of the trehalose-6-phosphate synthase gene *OsTPS1* enhances abiotic stress tolerance in rice. Planta.

[CR111] Liu R, Barkhordarian H, Emadi S, Park CB, Sierks MR (2005). Trehalose differentially inhibits aggregation and neurotoxicity of beta-amyloid 40 and 42. Neurobiol Dis.

[CR112] Liu Z, Ma C, Gao C, Xu P (2012). Efficient utilization of hemicellulose hydrolysate for propionic acid production using *Propionibacterium acidipropionici*. Bioresour Technol.

[CR113] Liu L, Zhuge X, Shin H, Chen RR, Li J, Du G, Chen J (2015). Improved production of propionic acid in *Propionibacterium jensenii* via combinational overexpression of glycerol dehydrogenase and malate dehydrogenase from Klebsiella pneumoniae. Appl Environ Microbiol.

[CR114] Louie G, Brownlie P, Lambert R, Cooper JB, Blundell TL, Wood SP, Warren MJ, Woodcock SC, Jordan PM (1992). Structure of porphobilinogen deaminase reveals a flexible multi domain polymerase with a single catalytic site. Nature.

[CR115] Lu P, Ma D, Chen Y, Guo Y, Chen GQ, Deng H, Shi Y (2013). L-glutamine provides acid resistance for *Escherichia coli* through enzymatic release of ammonia. Cell Res.

[CR116] Luggen AS (2006). Gerontologic nurse practicioner care guidelines: vitamin B12 deficiency in older adults. Geriat Nurs.

[CR117] Lyon W, Glatz B (1991). Partial purification and characterization of a bacteriocin produced by *Propionibacterium thoenii*. Appl Environ Microbiol.

[CR118] Lyon W, Sethi JK, Glatz B (1993). Inhibition of psychotrophic ogranisms by propionisin PLG-1, a bacteriocin produced by *Propionibacterium thoeni*. J Dairy Sci.

[CR119] Makihara F, Tsuzuki M, Sato K (2005). Role of trehalose synthesis pathways in salt tolerance mechanism of *Rhodobacter sphaeroides* f. sp. *denitrificans* IL106. Arch Microbiol.

[CR120] Mancini RJ, Lee J, Maynard HD (2011). Trehalose glycopolymers for stabilization of protein conjugates to environmental stressors. J Am Chem Soc.

[CR121] Mantere-Alhonen S (1995). Propionibacteria used as probiotics: a review. Lait.

[CR122] Marciset O, Jeronimus-Stratinh MC, Mollet B, Poolman B (1997). Thermophilin 13, a nontypical antilisterial poration complex bacteriocin, that functions without a receptor. J Biol Chem.

[CR123] Martens JH, Barg H, Warren MJ, Jahn D (2002). Microbial production of vitamin B12. Appl Microbiol Biotechnol.

[CR124] Marwaha S, Sethi R, Kennedy J (1983). Influence of 5,6dimethylbenzimidazole (DMB) on vitamin B12 biosynthesis by strains of *Propionibacterium*. Enzym Microb Technol.

[CR125] Meile L, Dasen G, Miescher S, Stierli M, Teuber M (1999). Classification of propionic acid bacteria and approaches to applied genetics. Lait.

[CR126] Meile L, Gwenaelle LB, Thierry A (2008). Safety assessment of dairy microorganisms: *Propionibacterium* and *Bifidobacterium*. Int J Food Microbiol.

[CR127] Meynial-Salles I, Dorotyn S, Soucaille P (2008). A new process for the continuous production of succinic acid from glucose at high yield, titer and productivity. Biotechnol Bioeng.

[CR128] Miescher SM, Stierli MP, Teuber M, Meile L (2000). Propionicin SM1, a bacteriocin from *Propionibacterium jensenii* DF1: isolation and characterization of the protein and its gene. Syst Appl Microbiol.

[CR129] Miks-Krajnik MH (2012). Rola paciorkowców mlekowych i pałeczek propionowych w procesie dojrzewania sera typu szwajcarsko-holenderskiego. ŻNTJ.

[CR130] Miura Y, You C, Ohnishi R (2008). Inhibition of Alzheimer amyloid β aggregation by polyvalent trehalose. Sci Tech of Adv Mat.

[CR131] Miyano K, Ye K, Shimizu K (2000). Improvement of vitamin B12 fermentation by reducing in the inhibitory metabolites by cell recycle system and mixed culture. J Biochem Eng.

[CR132] Moll GN, Konings WN, Driessen AJ (1999). Bacteriocins: mechanism of membrane insertion and pore formation. Anton Leeuw Int J G.

[CR133] Morgan SM, Galvin M, Kelly J, Sletten K, Hill C (1999). Development of a lacticin 3147-enriched whey powder with inhibitory activity against foodborne pathogens. J Food Protect.

[CR134] Murooka Y, Piao Y, Kiatpapan P, Yamashita M (2005). Production of tetrapyrrole compounds and vitamin B12 using genetically engineering of *Propionibacterium freudenreichii*. Lait.

[CR135] Nakano K, Kataoka H, Matsumara M (1996). High density culture of *Propionibacterium freudenreichii* coupled with propionic acid removal system with activated charcoal. J Ferment Bioeng.

[CR136] Neumann VC, Heath HE, LeBlanc PA, Sloan GL (1993). Extracellular proteolytic activation of bacteriolytic peptidoglycan hydrolases of Staphylococcus simulans biovar staphylolyticus. FEMS Microbiol Lett.

[CR137] Nielsen H, Engelbrecht S, Brunak S, von Heijne G (1997). Identification of prokaryotic and eukaryotic signal peptides and prediction of their cleavage sites. Protein Eng.

[CR138] Ortigues-Marty I, Thomas E, Prévéraud DP, Girard CL, Bauchart D, Durand D, Peyron A (2006). Influence of maturation and cooking treatments on the nutritional value of bovine meats: water losses and vitamin B12. Meat Sci.

[CR139] Othake S, Wang YJ (2010). Trehalose: current use and future applications. J Pharm Sci.

[CR140] Ousterout DG, Kabadi AM, Thakore PI, Majoros WH, Reddy TE, Gersbach CA (2014). Multiplex CRISPR/Cas9-based genome editing for correction of dystrophin mutations that cause Duchenne muscular dystrophy. Nat Commun.

[CR141] Paoloni-Giacombino A, Grimble R, Pichard C (2003). Genetics and nutrition. Clinic Nutr.

[CR142] Piao Y, Kiatpapan P, Yamashita M, Murooka Y (2004). Effects of expression of hemA and hemB genes on production of porphyrin in *Propionibacterium freudenreichii*. Appl Environ Microbiol.

[CR143] Piao Y, Yamashita M, Kawaraichi N, Asegawa R, Ono H, Murooka Y (2004). Production of vitamin B12 in genetically engineered *Propionibacterium freudenreichii*. J Biosci Bioeng.

[CR144] Piveteau P (1999). Metabolism of lactate and sugars by dairy propionibacteria: a review. Lait.

[CR145] Piwowarek K, Lipińska E (2015). Bakterie propionowe użyteczne w przemyśle spożywczym. Przem Spoż.

[CR146] Piwowarek K, Lipińska E, Hać-Szymańczuk E (2016). Possibility of using apple pomaces in the process of propionic-acetic fermentation. Electron J Biotechnol.

[CR147] Qu Q, Lee SJ, Boos W (2004). TreT, a novel Trehalose Glycosyltransferring synthase of the Hyperthermophilic Archaeon *Thermococcus litoralis*. J Biol Chem.

[CR148] Quesada-Chanto A, Afschar S, Wagner F (1994). Microbial production of propionic acid and vitamin B12 using molasses or sugar. Appl Microbiol Biotechnol.

[CR149] Ramsay J, Aly Hassan M, Ramsay B (1998). Biological conversion of hemicellulose to propionic acid. Enzym Microb Technol.

[CR150] Ran FA, Hsu PD, Wright J, Agarwala V, Scott DA, Zhang F (2013). Genome engineering usingt he CRISPR-Cas9 system. Nat Protoc.

[CR151] Ranadheera RDCS, Baines SK, Adams MC (2010). Importance of food in probiotic efficacy. Food Res Int.

[CR152] Ratnam P, Barefoot S, Prince L, Bodine A, McCaskill LH (1999). Partial purification and characterization of a bacteriocin produced by *Propionibacterium jenseni* B1264. Lait.

[CR153] Raux E, Lanois A, Levillayer F, Warren MJ, Brody E, Rambach A, Thermes C (1996). *Salmonella typhimurium* cobalamin (vitamin B12) biosynthetic genes: functional studies in *S. typhimurium* and *Escherichia coli*. J Bacteriol.

[CR154] Raux E, Lanois A, Rambach A, Warren A, Hermes C (1998). Cobalamin (vitamin B12) biosynthesis: identification and characterization of Bacillus megaterium cobI operon. Biochem J.

[CR155] Raux E, Schubert HL, Roper JM, Wilson KS, Warren MJ (1999). Vitamin B12: insights into biosynthesis’s mount improbable. Bioorg Chem.

[CR156] Reichardt N, Duncan S, Young P, Belenguer A, McWilliam Leitch C, Scott C, Flint H, Louis P (2014). Phylogenetic distribution of three pathways for propionate production within the human gut microbiota. ISME J.

[CR157] Rince A, Dufour A, Uguen P, le Pennec JP, Haras D (1997). Characterization of the lacticin 481 operon: the *Lactococcus lactis* genes lctF, lctE, and lctG encode a putative ABC transporter involved in bacteriocin immunity. Appl Environ Microbiol.

[CR158] Rodionov DA, Vitreschak AG, Mironov AA, Gelfand MS (2003). Comparative genomics of the vitamin B12 metabolism and regulation in prokaryotes. J Biol Chem.

[CR159] Roessner CA, Huang KX, Warren MJ, Raux E, Scott AI (2002). Isolation and characterization of 14 additional genes specifying the anaerobic biosynthesis of cobalamin (vitamin B12) of *Propionibacterium freidenreichii* (*P. shermani*). Microbiology.

[CR160] Roman RV, Iluc E, Mustea A, Neacsu A, Asandului V (2001). Optimisation of medium components in vitamin B12 biosynthesis. Roum Biotechnol Lett.

[CR161] Roth JR, Lawrence JG, Rubenfield M, Dieffer-Higgins S, Church GM (1993). Characterization of cobalamin (vitamin B12) biosynthetic genes of *Salmonella typhimurium*. J Bacteriol.

[CR162] Ruhal R, Aggarwal S, Choudhury B (2011). Suitability of crude glycerol obtained from biodiesel waste for the production of trehalose and propionic acid. Green Chem.

[CR163] Ruhal R, Choudhury B (2012a) Use of an osmotically sensitive mutant of *Propionibacterium freudenreichii* subsp. *shermanii* for the simultaneous productions of organic acids and trehalose from biodiesel waste based crude glycerol. Bioresour Technol 109:131–139. 10.1016/j.biortech.2012.01.039 10.1016/j.biortech.2012.01.039 22306074

[CR164] Ruhal R, Choudhury B (2012). Improved trehalose production from biodiesel waste using parent and osmotically sensitive mutant of *Propionibacterium freudenreichii* subsp. *shermanii* under aerobic conditions. J Ind Microbiol Biotechnol.

[CR165] Ruhal R, Kataria R, Choudhury B (2013). Trends in bacterial trehalose metabolism and significant nodes of metabolic pathway in the direction of trehalose accumulation. Microb Biotechnol.

[CR166] Ryan MP, Jack RW, Josten M, Sahl HG, Jung G, Ross RP, Hill C (1999). Extensive post-translational modification, including serine to D-alanine conversion, in the two-component lantibiotic, lacticin 3147. J Biol Chem.

[CR167] Ryu SI, Park CS, Cha J, Woo EJ, Lee SB (2005). A novel trehalose-synthesizing glycosyltransferase from *Pyrococcus horikoshii*: molecular cloning and characterization. Biochem Bioph Res Co.

[CR168] Santos F, Vera JL, Lamosa P, de Valdez GF, de Vos WM, Santos H (2007). Pseudovitamin B12 is the corrinoid produced by *Lactobacillus reuteri* CRL1098 under anaerobic conditions. FEBS Lett.

[CR169] Sattler I, Roessner CA, Stolowich NJ, Hardin SH, Haris-Haller LW, Yokubaitis NT, Murooka Y, Hashimoto Y, Scott AI (1995). Cloning, sequencing, and expression of the uroporphyrinogen III metyltransferase cobA gene of *Propionibacterium freidenreichii* (*shermani*). J Bacteriol.

[CR170] Schillinger U, Geisen R, Holzapfel WH (1996). Potential of antagonistic microorganisms and bacteriocins for the biological preservation of foods. Trends Food Sci.

[CR171] Scott AI (1994). The discovery of nature’s pathway to vitamin B12. A 25 year odyssey. Tetrahedron.

[CR172] Seidametova E, Shakirzyanova M, Ruzieva D, Gulyamova T (2004). Isolation of cobalt-resistant strains of propionic acid bacteria, potent producers of vitamin B12. Appl Biochem Biotechnol.

[CR173] Siegers K, Entian KD (1995). Genes involved in immunity to the lantibiotic nisin produced by *Lactococcus lactis* 6F3. Appl Environ Microbiol.

[CR174] Sip A, Krasowska M, Więckiewicz M (2009). Zastosowanie bakteriocyn klasy IIa bakterii fermentacji mlekowej. Biotechnologia.

[CR175] Smith AG, Croft MT, Moulin M, Webb ME (2007). Plants need their vitamins too. Curr Opin Plant Biol.

[CR176] Stjernholm R (1958). Formation of trehalose during dissimilation of glucose by *Propionibacterium*. ACTA Chem Scan.

[CR177] Strom AR, Kaasen (1993). Trehalose metabolism in *Escherichia coli*: stress protection and stress regulation of gene expression. Mol Microbiol.

[CR178] Suomalainen TH, Sigvart-Mattila P, Mättö J, Tynkkynen S (2008). In vitro and in vivo gastrointestinal survival, antibiotic susceptibility and genetic identification of *Propionibacterium freudenreichii* ssp. *shermanii* JS. Int Dairy J.

[CR179] Suwannakham S, Yang S (2005). Enhanced propionic acid fermentation by *Propionibacterium acidipropionici* mutant obtained by adaptation in a fibrous-bed bioreactor. Biotechnol Bioeng.

[CR180] Suwannakham S, Huang Y, Yang ST (2006). Construction and characterization of ack knock-out mutants of *Propionibacterium acidipropionici* for enhanced propionic acid fermentation. Biotechnol Bioeng.

[CR181] Teramoto N, Sachinvala ND, Shibata M (2008). Trehalose and trehalose-based polymers for environmentally benign, biocompatible and bioactive materials. Molecules.

[CR182] Thierry A, Maillard MB (2002). Production of cheese flavour compounds derived from amino acid catabolism by *Propionibacterium freudenreichii*. Lait.

[CR183] Thierry A, Maillard MB, Richoux R, Kerjean JR, Lortal S (2005). *Propionibacterium freudenreichii* strains quantitatively affect production of volatile compounds in Swiss cheese. Lait.

[CR184] Thirupathaiah Y, Swarupa Rani C, Sudhakara Reddy M (2012). Effect of chemical and microbial vitamin B_12_ analogues on production of vitamin B_12_. World J Microbiol Biotechnol.

[CR185] Trojanowska K, Czaczyk K (1996). The effect of Co++, Mg ++, Zn ++, Mn++, Fe++ ions on the course of propionic acid fermentation. Ann Univ Agric Poznan.

[CR186] Upreti GC, de Vuyst L, Vandamme EJ (1994). Bacteriocins of lactic acid bacteria.

[CR187] van der Merwe IR, Bauer R, Britz TJ, Dicks LMT (2004). Characterization of Thoeniicin 447, a bacteriocin isolated from *Propionibacterium thoenii* strain 447. Int J Food Microbiol.

[CR188] Van Luijk N, Stierli MP, Schwenninger SM, Hervé C, Dasen G, Jore JP, Pouwels PH, Van Der Werf MJ, Teuber M, Meile L (2002). Genetics and molecular biology of propionibacteria. Lait.

[CR189] Van Wyk J, Witthuhn RC, Britz TJ (2011). Optimisation of vitamin B12 and folate production by *Propionibacterium freudenreichii* strains in kefir. Int Dairy J.

[CR190] von Heijne G (1988). Transcending the impenetrable: how proteins come to terms with membranes. Biochim Biophys Acta.

[CR191] Wang P, Wang Y, Liu Y, Shi H, Su Z (2012). Novel in situ product removal technique for simultaneous production of propionic acid and vitamin B12 by expanded bed adsorption bioreactor. Bioresour Technol.

[CR192] Wang Z, Sun J, Zhang A, Yang ST, Yang ST, El-Enshasy HA, Thongchul N (2013). Propionic acid fermentation. Bioprocessing technologies in biorefinery for sustainable production of fuels, chemicals, and polymers.

[CR193] Wang Z, Yang ST (2013). Propionic acid production in glycerol/glucose co-fermentation by *Propionibacterium freudenreichii* subsp. *shermanii*. Bioresour Technol.

[CR194] Wang G, Abercrombie JG, Huang G (2014) Enhanced fed-batch production, partial purification, characterization of jenseniin P, and discovery of a new bacteriocin-like substance produced by *Propionibacterium jensenii* B1264. Eur Food Res Technol 239:79–86. 10.1007/s00217-014-2199-7

[CR195] Wang Z, Ammar EM, Zhang A, Wang L, Lin M, Yang ST (2015b) Engineering *Propionibacterium freudenreichii* subsp*.**shermanii* for enhanced propionic acid fermentation: effects of overexpressing propionyl-CoA:succinate CoA transferase. Metab Eng 27:46–56. 10.1016/j.ymben.2014.10.005 10.1016/j.ymben.2014.10.005 25447642

[CR196] Wang Z, Lin M, Wang L, Ammar EM, Yang ST (2015a) Metabolic engineering of *Propionibacterium freudenreichii* subsp. *shermanii* for enhanced propionic acid fermentation: effects of overexpressing three biotin-dependent carboxylases. Process Biochem 50:194–204. 10.1016/j.procbio.2014.11.012 10.1016/j.ymben.2014.10.005 25447642

[CR197] Wang P, Zhang Z, Jiao Y, Liu S, Wang Y (2015) Improved propionic acid and 5,6-dimethylbenzimidazole control strategy for vitamin B12 fermentation by *Propionibacterium freudenreichii*. J Biotechnol 193:123–129. 10.1016/j.jbiotec.2014.11.019 10.1016/j.jbiotec.2014.11.019 25455014

[CR198] Warren MJ, Roessner CA, Santander PJ, Scott AI (1990). The Escherichia coli cysG gene encodesS-adenosyl-methionine-dependent uroporphyrinogen III methylase. Biochem J.

[CR199] Warren MJ, Cooper JB, Woods SP, Shoolingin-Jordan PM (1998). Lead poisoning, haem synthesis and 5-aminolaevulinic acid dehydratase. Trends Biochem Sci.

[CR200] Warren MJ, Raux E, Schubert HL, Escalante-Semerena JC (2002). The biosynthesis of adenosylcobalamin (vitamin B12). Nat Prod Rep.

[CR201] Wei P, Lin M, Wang Z, Fu H, Yang H, Jiang W, Yang ST (2016). Metabolic engineering of *Propionibacterium freudenreichii* subsp. *shermanii* for xylose fermentation. Bioresour Technol.

[CR202] Wiedenheft B, Sternberg SH, Jennifer A, Doudna JA (2012). RNA-guided genetic silencing systems in bacteria and archaea. Nature.

[CR203] Weinbrenner DR, Barefoot SF, Grinstead DA (1997). Inhibition of yoghurt starter cultures by jenseniin G, a *Propionibacterium* bacteriocins. J Dairy Sci.

[CR204] Wolf A, Krämer R, Morbach S (2003). Three pathways for trehalose metabolism in *Corynebacterium glutamicum* ATCC13032 and their significance in response to osmotic stress. Mol Microbiol.

[CR205] Yang ST, Huang Y (1995). A novel recycle batch immobilized cell bioreactor for propionate production from whey lactose. Biotechnol Bioeng.

[CR206] Yazdani SS, Gonzales R (2007). Anaerobic fermentation of glycerol: a path to economic viability for the biofuels industry. Curr Opini Biotech.

[CR207] Yu WB, Jiang T, Lan DM, Lu JH, Yue ZY, Wang J, Zhou P (2012). Trehalose inhibits fibrillation of A53T mutant alpha-synuclein and disaggregates existing fibrils. Arch Biochem Bioph.

[CR208] Yu Y, Zhu X, Shen Y (2015). Enhancing the vitamin B12 production and growth of *Propionibacterium freudenreichii* in tofu wastewater via a light-induced vitamin B12 riboswitch. Appl Microbiol Biotechnol.

[CR209] Zang C, Brandt M, Schwab C, Ganzle M (2010). Propionic acid production by cofermentation of *Lactobacillus buchneri* and *Lactobacillus diolivorans* in sourdough. Food Microbiol.

[CR210] Zárate G, Pérez-Chaia A, Oliver G (2002). Some characteristics of practical relevance of the α-galactosidase from potential probiotic strains of *Propionibacterium acidipropionici*. Anaerobe.

[CR211] Zhang ST, Matsuoka H, Toda K (1993). Production and recovery of propionic and acetic acid in electrodialysis culture of *Propionibacterium shermani*. J Ferment Bioeng.

[CR212] Zhang A, Yang ST (2009). Engineering *Propionibacterium acidipropionici* for enhanced propionic acid tolerance and fermentation. Biotechnol Bioeng.

[CR213] Zhu Y, Li J, Tan M, Liu L, Jiang L, Sun J, Lee P, Du G, Chen J (2010). Optimization and scale-up of propionic acid production by propionic acid-tolerant *Propionibacterium acidipropionici* with glycerol as the carbon source. Bioresour Technol.

[CR214] Zhu L, Wei P, Cai J, Zhu X, Wang Z, Huang L, Xu Z (2012). Improving the productivity of propionic acid with FBB-immobilized cells of an adapted acid-tolerant *Propionibacterium acidipropionici*. Bioresour Technol.

